# Source‐Independent Enrichment of Light Lanthanides: Microbial Mobilization, Selective Uptake, and Intracellular Storage

**DOI:** 10.1111/1462-2920.70413

**Published:** 2026-09-16

**Authors:** Linda Gorniak, Sophie M. Gutenthaler‐Tietze, Alina Lobe, Lena J. Daumann, Robin Steudtner, Thorsten Schäfer, Frank Steiniger, Martin Westermann, Kirsten Küsel, Carl‐Eric Wegner

**Affiliations:** ^1^ Institute of Biodiversity, Aquatic Geomicrobiology, Friedrich Schiller University Jena Germany; ^2^ Applied Geology, Institute of Geosciences, Friedrich Schiller University Jena Germany; ^3^ Chair of Bioinorganic Chemistry, Heinrich Heine University Düsseldorf Germany; ^4^ Department of Chemistry Ludwig Maximilian University München Germany; ^5^ Institute of Resource Ecology, Helmholtz‐Zentrum Dresden‐Rossendorf Dresden Germany; ^6^ University Hospital Jena, Electron Microscopy Center Jena Germany; ^7^ Cluster of Excellence Balance of the Microverse, Friedrich Schiller University Jena Germany; ^8^ German Center for Integrative Biodiversity Research (iDiv) Halle‐Jena‐Leipzig Germany; ^9^ Chair of Bioinorganic Chemistry, Biomics Group, Heinrich Heine University Düsseldorf Germany

**Keywords:** electron microscopy, elemental analysis, lanthanides, lanthanome, RNAseq

## Abstract

Poorly soluble lanthanide minerals are a challenge for the sustainable extraction of lanthanides as key resources for the green energy transition and lanthanide‐dependent microbial metabolism. Lanthanide‐utilising bacteria are widespread in the environment. Their preference for light lanthanides requires differentiation mechanisms that enable downstream utilisation. Whether lanthanide discrimination occurs during access, mobilisation, uptake, or intracellular processing is mostly unknown and likely controlled by habitat and bioavailability. Using *Beijerinckiaceae* bacterium RH AL1 and combining transcriptomics, analytics, and electron microscopy, we studied microbial lanthanide mobilisation and uptake from different lanthanide minerals, an alloy, and pure lanthanide compounds. Strain AL1 is a facultative methylotroph that depends on light lanthanides for methanol oxidation and known for periplasmic lanthanide accumulation. We could show that strain AL1 grew with all tested lanthanide sources and selectively enriched light lanthanides independent of the source, lanthanide content, and the proportion of light lanthanides. Transcriptomics revealed that the source type significantly influenced gene expression. Lanthanide discrimination in *Beijerinckiaceae* bacterium RH AL1 is a multilayered process, likely rooted in the complementary action of chelation, uptake, and periplasmic storage. Strains that mobilise lanthanides and increase their bioavailability can transform mineral‐bound lanthanides into shared resources for microbial communities, with implications for sustainable lanthanide use.

## Graphical abstract

Only light lanthanides facilitate lanthanide‐dependent methylotrophy. In *Beijerinckiaceae* bacterium RH AL1, light lanthanides are enriched from diverse sources, including poorly soluble minerals, through a multilayered process involving mobilisation, selective uptake, and intracellular periplasmic storage.

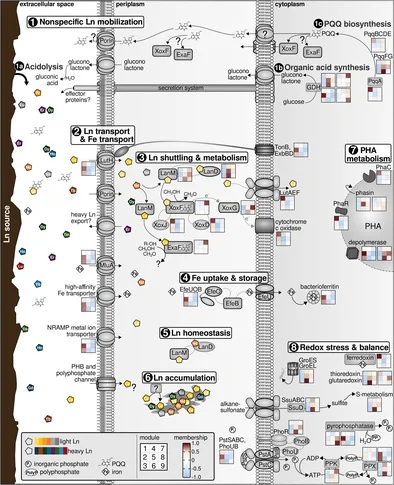

## 1 Introduction

Lanthanides (Lns, lanthanide [Ln]), the f‐elements comprising the elements La‐Lu (Table S1), are classified as critical resources (European Commission 2023) and are key for the ongoing green energy transition (Balaram 2019). Although abundant in nature, enriched, economically profitable deposits are rare. Moreover, Lns co‐occur, and their overall similar characteristics make complex and cost‐intensive separation and refinement processes necessary. Lns play an important role in microbial carbon cycling, especially in C_1_‐metabolism, and likely have broader roles in microbial metabolism (Wehrmann et al. 2017, 2020; Gorniak et al. 2023, 2024). We are only beginning to unravel how microbes mobilise, take up, and distinguish between Lns, all of which can facilitate sustainable Ln use.

Known biomolecules directly involved in Ln sensing, utilisation, and transport (summarized as the lanthanome (Cotruvo Jr 2019; Mattocks et al. 2019)) include the Ln‐binding proteins lanmodulin (LanM) (Cotruvo Jr et al. 2018), lanpepsy (Hemmann et al. 2023), landiscernin (LanD) (Larrinaga et al. 2024), as well as the Ln‐utilisation and transport (*lut*‐) (Cotruvo Jr et al. 2018; Ochsner et al. 2019; Roszczenko‐Jasińska et al. 2020) and methylolanthanin (a Ln‐binding metallophore) uptake (*mlu*−/*mll*‐) clusters (Zytnick et al. 2024). Lanmodulin attracted significant attention, enabling the development of a high‐sensitivity Ln sensor and a pilot‐scale platform for selective Ln recycling (Mattocks et al. 2019; Dong et al. 2021). Known Ln‐dependent enzymes rely on pyrroloquinoline quinone (PQQ) as a cofactor and function as alcohol dehydrogenases (ADH) (Keltjens et al. 2014). Ln‐dependent Xox‐type methanol dehydrogenases (MDH) represent key enzymes for methanol oxidation in gram‐negative bacteria and are arguably ecologically more relevant than their calcium‐dependent counterparts. Transcript abundances of *xoxF* significantly exceed those encoding Ca‐dependent Mxa‐type MDHs in the surface ocean (Voutsinos et al. 2025). Recent findings suggest that Ln‐ and PQQ‐dependent proteins are involved in diverse carbon substrate metabolism (Voutsinos et al. 2026).

Lns are commonly divided into light (La‐Eu) and heavy (Gd‐Yb) Lns (Gonzalez et al. 2014; Kotelnikova et al. 2021). Ln‐dependent microbial growth is primarily facilitated by light Lns (Daumann 2019). Heavy Lns have been observed to be taken up and stored (Gorniak et al. 2023), but no function in microbial metabolism has yet been assigned to them. It is currently unclear if discrimination between light and heavy Lns occurs during mobilisation, uptake, or intracellular processing. Studies investigating Ln‐dependent metabolism have mainly used soluble Ln salts, often at higher concentrations, which only distantly mimic natural conditions. Lns are typically present as poorly soluble minerals, including monazite and xenotime (phosphate minerals), as well as bastnaesite (a carbonate mineral) (Banfield and Eggleton 1989; Firsching and Brune 1991; Taunton et al. 2000; Zaharescu et al. 2017), which are primary Ln sources for mining (U.S. Geological Survey 2025).

Ln bioavailability depends on the environment. Mechanisms of Ln mobilisation, discrimination, and uptake/storage likely differ across taxa depending on habitat. In this study, we chose *Beijerinckiaceae* bacterium RH AL1 (strain AL1) to investigate the mobilisation of Lns from (i) Ln‐containing minerals and the alloy ferrocerium, as well as (ii) poorly soluble Ln forms (La_2_O_3_, Nd_2_O_3_, LaPO_4_). Different mineral types with different Ln contents and proportions of light and heavy Ln elements were tested, including apatite, monazite, and xenotime (phosphate minerals), bastnaesite (a carbonate), loparite (an oxide), and gadolinite (a silicate). The tested minerals differed by recalcitrance, which we define here as the difficulty with which Lns can be accessed. AL1 was previously isolated from early‐industrial soft‐coal slags (Wegner and Liesack 2017; Wegner et al. 2020). It is a facultative methylotroph that naturally depends on light Lns for methanol oxidation utilisable Lns: La—Nd (Wegner et al. 2020), Sm and Eu have not been tested. It contains a single *xoxF* gene in its genome and no *mxaF* homologue. The genome also features an *exaF* (coding for a broad‐substrate alcohol dehydrogenase) and PQQ ADH 9 gene (Wegner et al. 2020). The strain is of particular interest for biotechnological applications, as it has previously been shown to take up Lns and form periplasmic Ln accumulations located at the cell poles, near polyhydroxyalkanoate (PHA) granules (Wegner et al. 2021; Gorniak et al. 2023). We were interested in whether Ln mobilisation, uptake, and intracellular storage are selective processes, and at which stage discrimination between different Lns occurs. Mobilisation and increased Ln bioavailability are required for Lns to play a broader role in microbial communities and microbial physiology.

## 2 Experimental Procedures

### 2.1 Sourcing Ln‐Containing Minerals and Ferrocerium

The minerals have been obtained through colleagues (bastnaesite [origin: Nam Xe, Vietnam; 1983], loparite [USSR, 1988], gadolinite [Norway, 1958], apatite [Finland, Kola Peninsula, Institute of Chemistry and Technology of Rare Elements and Mineral Resources; 1985]) and retailers (xenotime and monazite [Etsy, TheGlobalStone]. Ferrocerium was donated [Treibacher Industrie AG, Althofen, Austria]).

### 2.2 Analytical Investigation of Ln Sources

Elemental analysis (C, H, N, S) was performed at the analytical facility of the Department of Chemistry and Pharmacy, Ludwig Maximilian University of Munich (LMU), Germany, using a Vario EL cube instrument (Elementar Analysensysteme GmbH, Langenselbold, Germany). Inductively coupled plasma mass spectrometry (ICP‐MS) was performed on milled and acid‐digested (Table S2) minerals at the analytical facility of the Helmholtz Centre Dresden‐Rossendorf, Germany, using an iCAP RQ ICP‐MS device (Thermo Fisher Scientific, Frankfurt, Germany). More information is provided in the Supporting Information. Mineral compositions were verified by scanning electron microscopy (SEM) coupled to energy‐dispersive X‐ray spectroscopy (EDX) with a Helios Nanolab G3 Dual Beam UC (FEI, part of ThermoFisher Scientific, Darmstadt, Germany) coupled to an X‐Max 80 SDD detector (Oxford Instruments GmbH, Wiesbaden, Germany). Measurements were conducted at the Analytic Facility of the Department of Chemistry and Pharmacy at LMU.

### 2.3 Cultivation

Cultivations with *Beijerinckiaceae* bacterium RH AL1 were done as outlined before (Gorniak et al. 2023) in MM2 medium (Dedysh et al. 2012) with methanol as the carbon source (123 mM, 0.5% [v/v]) and 0.1% [v/v] of trace element solution no. 1 (Dedysh and Dunfield 2014). Two sets of incubations (Table S3) were done with each condition (= different Ln source) in triplicates. For the first set, Ln‐containing minerals (apatite, bastnaesite, gadolinite, loparite, monazite, or xenotime; 5 mg [milled] mineral per culture [140 mL], 0.26–16.14 μmol Ln) or the alloy ferrocerium (one piece of 145.27 ± 2.48 mg per culture [140 mL], 770.55 μmol Ln) (Table S4) served as Ln sources. For the second, La or Nd (1 μM) were supplied in different forms: as (i) chlorides, (ii) oxides, or (iii) phosphate (La only). RNA extraction samples were collected during late exponential growth. Cells were harvested and stored at −80°C until further processing. Controls with methanol, without added Lns, were run in parallel to detect Ln leaching from glassware and carbon‐source carryover from pre‐cultures. Additional controls with Ln minerals or ferrocerium, but without methanol, were used to verify that AL1 did not utilise carbon from the Ln sources. More information is provided in the Supporting Information. Growth metrics were calculated using *growthcurver* (v0.3.1) (Sprouffske and Wagner 2016). Significant differences regarding obtained growth rates were identified using the Student's *t*‐test implemented in base *R*.

### 2.4 Elemental Analysis of Supernatant Samples

Supernatant analytics were carried out to assess the solubility of the tested lanthanide sources dependent on pH and to probe for lanthanide depletion/uptake during cultivation. Supernatant samples were collected from biotic samples during biomass harvest for downstream RNAseq analysis. We carried out abiotic incubations in MM2 medium adjusted to pH 5 or 3 to mimic the pH decrease observed during biotic incubations. We matched sampling time points from those to the biotic incubations. Elemental analysis of filtered (0.22 μm PES) supernatant samples was done with an 8900 Triple Quadrupole ICP‐MS device (Agilent Technologies, Waldbronn, Germany) coupled to an autosampler prepFAST4DX System (Elemental Scientific, Mainz, Germany).

### 2.5 Electron Microscopic Investigations of Biomass Samples

Biomass samples from replicated incubations and matching timepoints (Figure S1) were collected via centrifugation (2 ✕ [10 min., 10,000 ✕ g at room temperature]). Sample preparation and subsequent analysis through transmission electron microscopy (TEM) and EDX were done as described previously (Wegner et al. 2021; Gorniak et al. 2023). EDX spectra have been deconvoluted using the Quantax software (Bruker, Berlin, Germany).

### 2.6 Calculation of Ln Enrichment Factors

Enrichment factors (EFs) have been calculated for light (La‐Eu), utilisable (La‐Nd), and heavy (Gd‐Lu) lanthanides (Table S5). Element‐specific EFs have been calculated as well (Table S6). We distinguished two stages regarding Ln enrichment: source → supernatant (mobilisation/dissolution), and supernatant → periplasmic deposits (uptake/storage). Details regarding the procedure can be found in the Supporting Information.

### 2.7 RNA Extraction, mRNA Enrichment, Library Preparation, and Sequencing

Late‐exponential biomass samples were subjected to total RNA extraction, mRNA enrichment, and RNAseq library preparation as previously described (Wegner et al. 2021; Gorniak et al. 2023). Sequencing libraries were equimolarly pooled. Illumina sequencing (2 ✕ 100 bp, paired‐end) on a NovaSeq 6000 instrument with an SP flowcell (Illumina, San Diego, California, USA) was performed by the sequencing core facility of the Leibniz Institute on Aging—Fritz Lipmann Institute (Jena, Germany).

### 2.8 Sequence Data Pre‐Processing and Differential Gene Expression Analysis

The quality of raw and trimmed sequences was checked with *fastQC* (v0.11.9) (Andrews 2010). Data pre‐processing has been outlined before (Gorniak et al. 2023, 2024), and is covered in the Supporting Information, including pseudocount distributions (Figure S2), biological coefficients of variation (Figure S3), and *p*‐value distributions regarding differential gene expression analysis (Figure S4). Differential gene expression analysis (DGEA) was performed using the R software environment (v.4.2.1) (R Core Team 2022) and the package *edgeR* (v.3.42.4) (Robinson et al. 2010) together with *limma* (v3.50.0) (Ritchie et al. 2015), *mixOmics* (v6.18.1) (Rohart et al. 2017), *HTSFilter* (v1.34.0) (Rau et al. 2013), and *bigPint* (v1.10.0) (Rutter and Cook 2020). Genes featuring changes in gene expression above |0.58| log_2_FC (fold change), gene expression values higher than 4 (log_2_CPM, counts per million), and FDR (false discovery rate)‐adjusted *p*‐values below 0.05 were considered for further analysis.

### 2.9 Weighted Gene Correlation Network Analysis ( *WGCNA* ) and Permutational Multivariate Analysis of Variance ( *PERMANOVA* )

Pre‐processed RNAseq data were filtered based on expression (log_2_CPM values > 4) and normalised with *edgeR* (Figure S5). Co‐expression networks were generated using the WGCNA package (v.1.73) (Langfelder and Horvath 2008; Langfelder and Horvath 2012), including its relevant dependencies. A soft‐threshold power of 15 was chosen for network construction based on the scale‐free fit index and mean connectivity across various soft‐thresholding powers (Figure S6). Genes with similar expression patterns were grouped into nine modules (Figure S7), and correlations between module eigengenes and selected physiological traits were calculated. FDR‐corrected *p*‐values were determined by applying the Benjamini‐Hochberg procedure (Benjamini and Hochberg 1995). We tested whether gene expression changes can be linked to covariates such as iron content, Ln availability, and observed differences in growth rather than the identity of the Ln sources themselves (Table S7). We performed a sequential permutational multivariate analysis of variance (PERMANOVA) on Euclidean distances calculated from standardised (z‐scored) log_2_CPM values using the *adonis2* function implemented in *vegan* (v2.7.5) (Oksanen et al. 2017). The procedure is described in more detail in the Supporting Information.

### 2.10 Figure Generation

Figures have been generated using the R software framework (v4.2.1), taking advantage of the following packages: *ggplot2* (v3.3.6) (Wickham 2011), *gplots* (v3.3.0) (Warnes et al. 2025), *ggpubr* (v0.4.0) (Kassambara 2018), *cowplot* (v1.1.1) (Wilke 2019), and *upsetR* (v1.4.0) (Conway et al. 2017), including their respective dependencies. Figures have been finalised with *inkscape* (https://inkscape.org/).

## 3 Results

### 3.1 Characterisation of Different Ln Minerals

**FIGURE 1:**
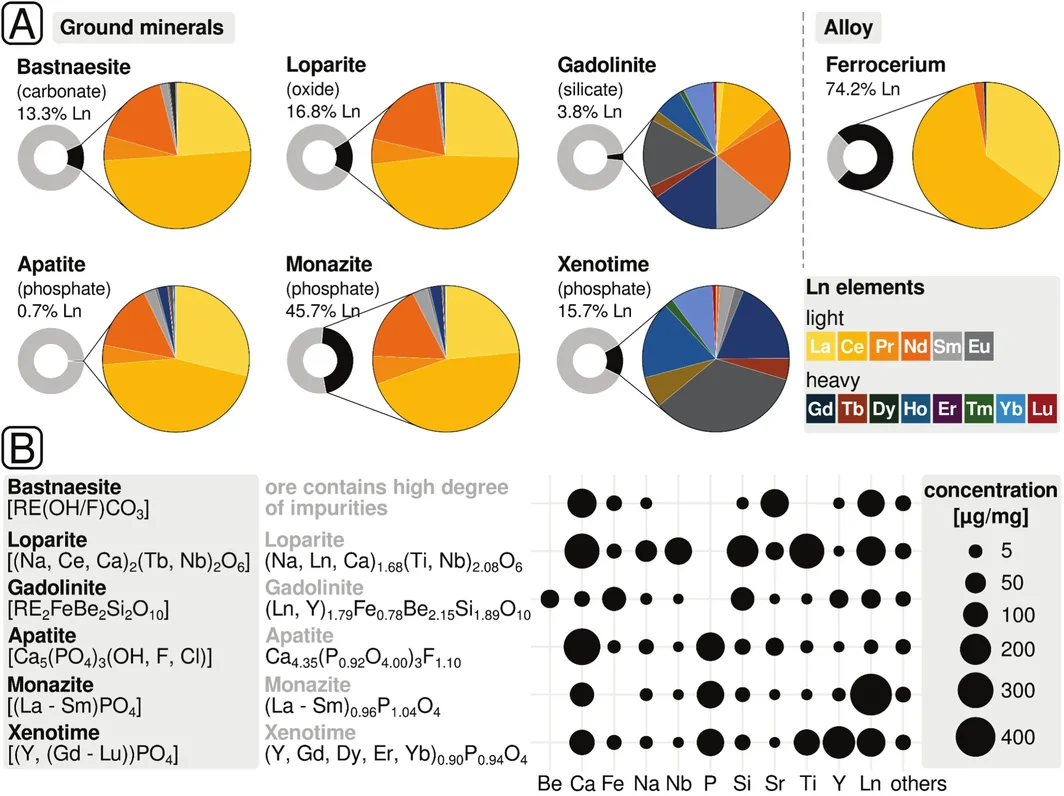
Characterisation of Ln‐containing minerals and the alloy ferrocerium. Lns present in the investigated Ln sources were determined via ICP‐MS (A), and sum formulas (Miyawaki et al. 1984; Foerster 1998; Williams et al. 2002; Pasero et al. 2010; Shivaramaiah et al. 2016) and element ratios deduced (B) based on EDX and ICP‐MS measurements. Concentrations of detected elements measured via ICP‐MS are shown as well. More detailed information about mineral/alloy compositions based on EDX (Table S8) and ICP‐MS (Table S9) can be found in the Supporting Information. RE = Rare Earth (Table S1).

We examined different types of Ln‐containing minerals, including phosphates (apatite, monazite, xenotime), an oxide (loparite), a silicate (gadolinite), and a carbonate (bastnaesite), through EDX and ICP‐MS (Figure 1A), before using them as Ln sources in incubation experiments. The determined elemental compositions matched the respective sum formulas, with varying degrees of impurity (Figure 1B), based on EDX (Table S8) and ICP‐MS (Table S9). Monazite featured the highest Ln content (Figure 1A+B) among the selected minerals (457.39 μg/mg Ln). Lower Ln concentrations were detected in loparite, xenotime, and bastnaesite, ranging from 132.64 μg/mg (bastnaesite)–168.10 μg/mg (loparite). Only 38.07 and 7.429 μg/mg Lns were detected in gadolinite and apatite. We also considered the alloy ferrocerium as a Ln source, which was primarily composed of cerium (47.1% ± 0.2%), lanthanum (26.7% ± 0.8%), and iron (21.3% ± 0.9%), as determined by ICP‐MS (Table S9). An elevated iron content was also observed in gadolinite (87.2 μg/mg), consistent with its stoichiometry (Figure 1B). Phosphorus was only present in apatite, monazite, and xenotime (135–156 μg/mg), matching their classification as phosphate minerals, and was below the detection limit in the other sources (Table S9). Strain AL1 was previously shown to require light Lns for methylotrophic growth (utilisable Lns: La—Nd (Wegner et al. 2020), Sm and Eu have not been tested). La—Nd made up the majority of Lns (92.5% –99.3%) in most minerals (monazite, bastnaesite, loparite, and apatite) and ferrocerium, with Ce (44.9%–62.0% of total Ln) and La (23.5%–35.2% of total Ln) being especially abundant. Gadolinite and xenotime contained more heavy Lns.

### 3.2 Methylotrophic Growth in Response to Different Ln Sources

**FIGURE 2:**
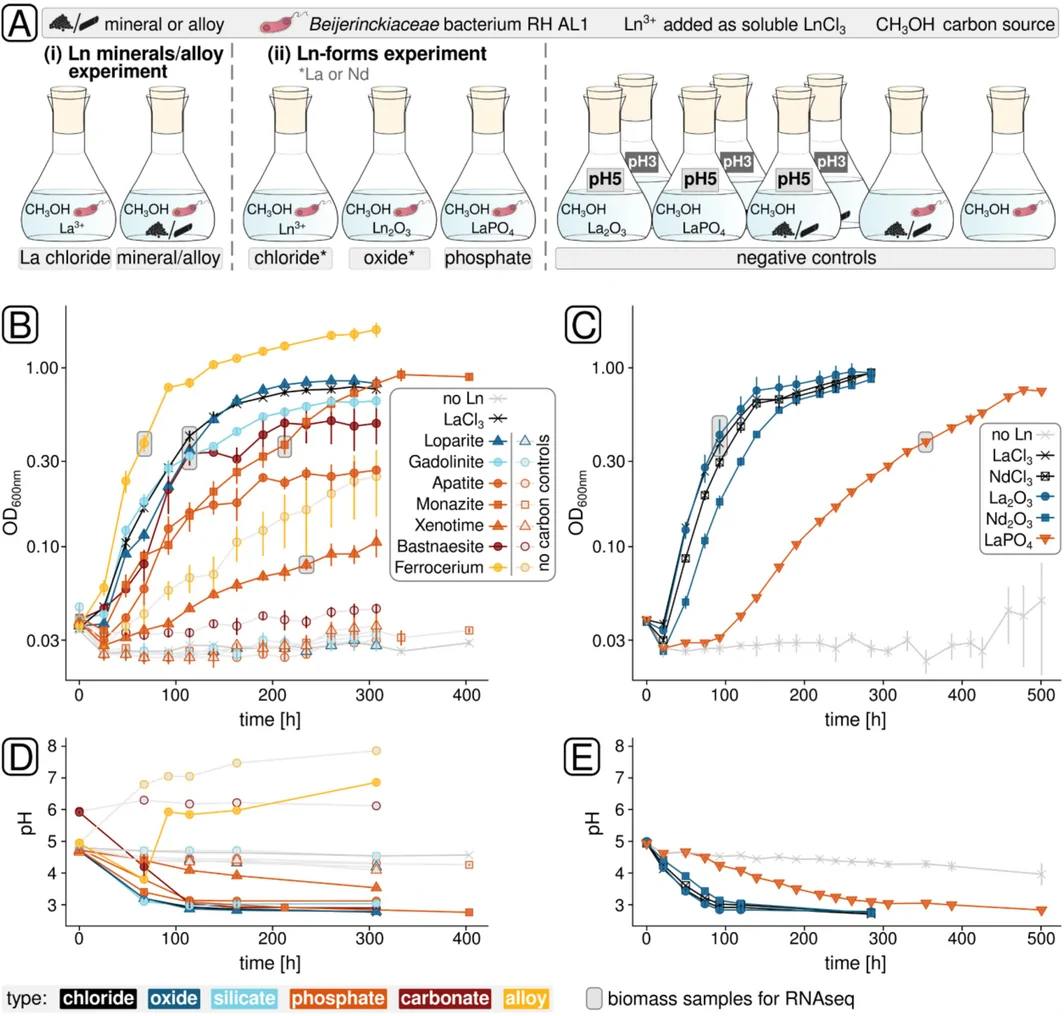
Methylotrophic growth of *Beijerinckiaceae* bacterium RH AL1 with the different tested Ln sources. In two growth experiments, the effects of (i) Ln‐containing minerals or the alloy ferrocerium and (ii) Ln forms (chloride vs. oxide vs. phosphate) were tested (A). Incubations were done in triplicates. Abiotic controls were prepared in two sets with MM2 medium adjusted to pH 5 or pH 3 (reflecting the initial pH used for cultivation and the pH drop observed during cultivation). Growth was either assessed based on incubations with different Ln minerals and ferrocerium (B), or different forms of La and Nd (C) as Ln sources. In addition to optical density measurements, we monitored the pH over the course of the incubations (D + E). Grey boxes highlight the time points at which biomass samples for RNAseq and supernatant samples for analytics were collected. OD = optical density.

The ability of strain AL1 to utilise different Ln sources for growth on methanol (0.5% [v/v], 123 mM) was tested in two cultivation experiments (Figure 2A, Table S3). All tested Ln sources facilitated growth (Figure 2B+C). LaCl_3_, Ln_2_O_3_, and loparite showed comparable growth patterns (Table S10), with growth rates (μ) ranging between 0.028 ± 0.004 h^−1^ (LaCl_3_) and 0.035 ± 0.004 h^−1^ (La_2_O_3_). Gadolinite addition led to a slightly lower growth rate (0.022 ± 0.002 h^−1^) and lower optical densities (OD_600nm_ 0.653 ± 0.019) compared to soluble LaCl_3_ (OD_600nm_ 0.761 ± 0.048). With the exception of apatite (0.028 ± 0.005 h^−1^), we observed significantly (Student's *t*‐test, *p* < 0.05) lower growth rates for cultures supplied with Ln phosphates (0.012 ± 0.001 h^−1^ [LaPO_4_], 0.016 ± 0.002 h^−1^ [xenotime], 0.019 ± 0.001 h^−1^ [monazite]). Apatite cultures became stationary early at an OD_600nm_ of 0.26 ± 0.02. The highest growth rate (0.036 ± 0.005 h^−1^) and final optical density (OD_600nm_ 1.324 ± 0.063) were observed with ferrocerium. Abiotic controls supplied with ferrocerium slightly increased in optical density, which was attributed to a reaction between the alloy and the medium.

All cultures, except those supplemented with bastnaesite, had an initial pH of 4.7–5.0. During exponential growth, the pH decreased to 2.8–3.1 until the cultures entered the stationary phase (Figure 2D+E). Incubations with ferrocerium were characterised by a decrease in pH during the first 3 days of cultivation, followed by an increase accompanied by a colour change to orange‐brown between 67 and 92 h of cultivation. Abiotic controls showed a stable pH over time, except for ferrocerium.

### 3.3 (A)biotic Dissolution and Intracellular Ln Accumulation

**FIGURE 3:**
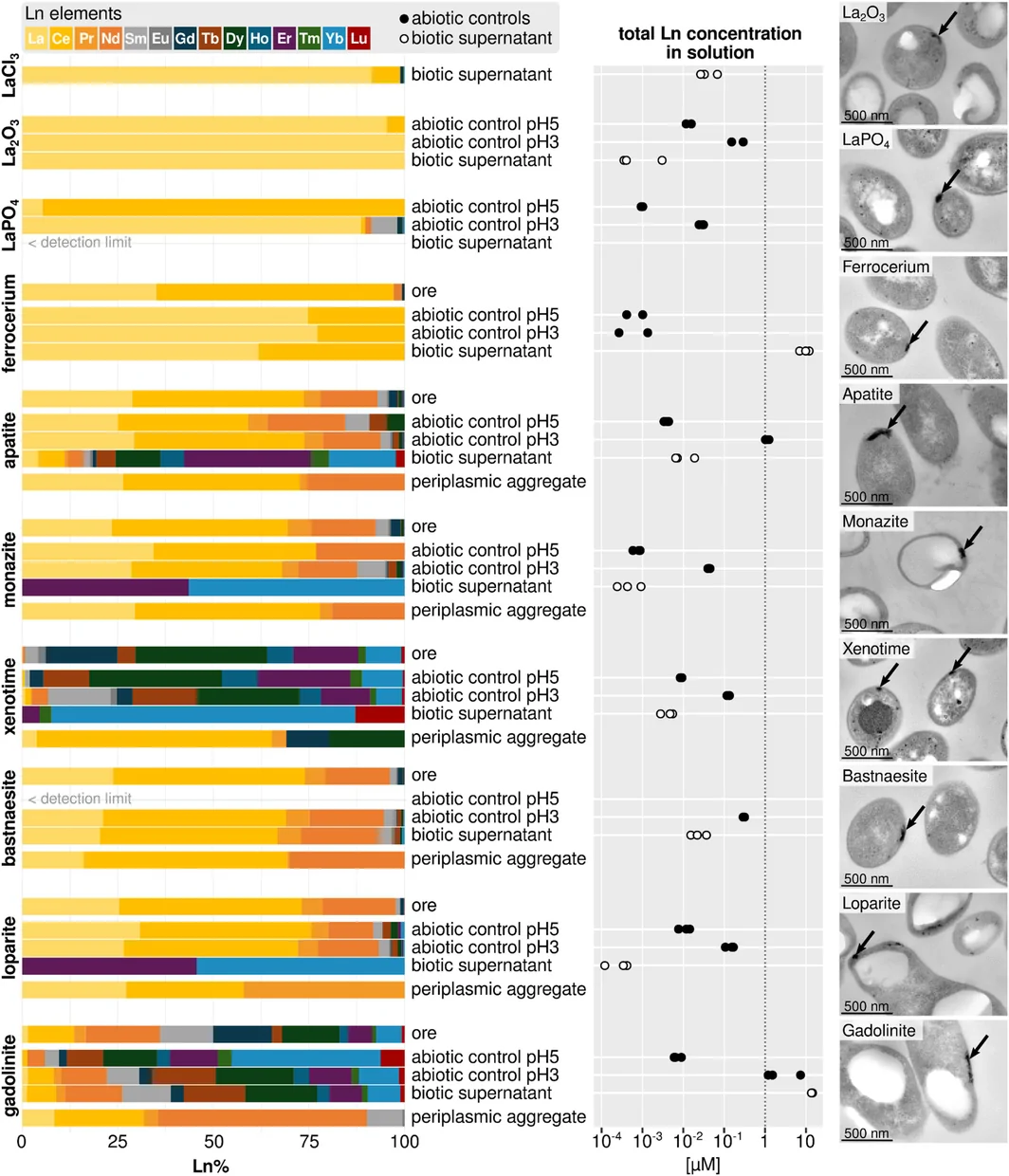
Intracellular Ln accumulation and (a)biotic dissolution. Intracellular Ln accumulation was verified through transmission electron microscopy (TEM). Periplasmic Ln accumulations are highlighted through black arrows. The abundance of Lns in (a)biotic supernatants and periplasmic deposits has been determined by ICP‐MS and EDX, respectively. Ln proportions in the Ln sources are the same as in Figure 1A. The molarity was deduced from the ICP‐MS data and the used cultivation volume. The previously determined optimum concentration for growth with soluble Ln chlorides (1 μM La (Wegner et al. 2020)) is highlighted for orientation. Supernatant analytics was performed in biological and technical replicates. EDX analysis was based on technical triplicates, the TEM micrographs show representative periplasmic deposits.

Incubations without strain AL1 at pH 5 and pH 3 allowed us to assess abiotic Ln dissolution (Figure 3, Table S11). We detected less than 1.42 × 10^−2^ μM Lns in any supernatant sample at pH 5. Aside from bastnaesite samples, which contained no detectable Ln, the lowest Ln concentrations were detected in ferrocerium (4.74 × 10^−4^ μM Ln) incubations. At pH 3, the minerals were more prone to dissolution. The highest Ln concentrations were detected in supernatant samples from gadolinite (3.35 μM Ln) and apatite (1.11 μM Ln) incubations. In these two cases, 37.9 (gadolinite) and 59.0% (apatite) of the originally added Lns ended up in solution (Table S12). Detected Ln concentrations were substantially lower for ferrocerium (5.36 × 10^−4^ μM Ln) and the Ln phosphates monazite and xenotime (≤ 1.27 × 10^−1^ μM Ln). The proportion of dissolved Lns was orders of magnitude smaller and did not exceed 0.89% (bastnaesite). In comparison, supernatant samples from AL1 cultures showed lower Ln concentrations (≤ 2.46 × 10^−2^ μM Ln), except for gadolinite (14.98 μM Ln) and ferrocerium (9.56 μM Ln). Unlike abiotic controls, samples from cultures grown with loparite or phosphate minerals were depleted in light Ln elements.

*Beijerinckiaceae* bacterium RH AL1 has previously been shown to form periplasmic Ln deposits when cultivated with soluble Ln chlorides (Wegner et al. 2021; Gorniak et al. 2023). We observed periplasmic deposits by TEM for all Ln sources (Figure 3). EDX (Table S13) confirmed mineral‐like Ln accumulations for cultures grown with Ln‐containing phosphate minerals (Figure S8) and those grown with bastnäsite, loparite, and gadolinite (Figure S9). Even cells grown with minerals containing little Lns overall or with a Ln content dominated by heavy Lns showed primarily light Lns in biominerals (Figure 3). Detected element ratios indicated that Lns are accumulated and stored as Ln phosphates.

We calculated Ln enrichment factors (EFs) for light, utilisable, and heavy Lns (Table S5), but also element‐specific EFs (Table S6) for the periplasmic deposits, relative to the respective Ln source. Enrichment of light and utilisable Lns was negligible for sources dominated by light lanthanides. It reached up to 10‐ and 74‐fold, respectively, for xenotime, which is dominated by heavy Lns, and was more pronounced regarding uptake and storage than during mineral dissolution. Since several of these EFs are based on Ln concentrations at or near the analytical detection limit, they should only be considered as evidence for the direction and relative degree of enrichment.

### 3.4 Differential Gene Expression in Response to Different Ln Sources

**FIGURE 4:**
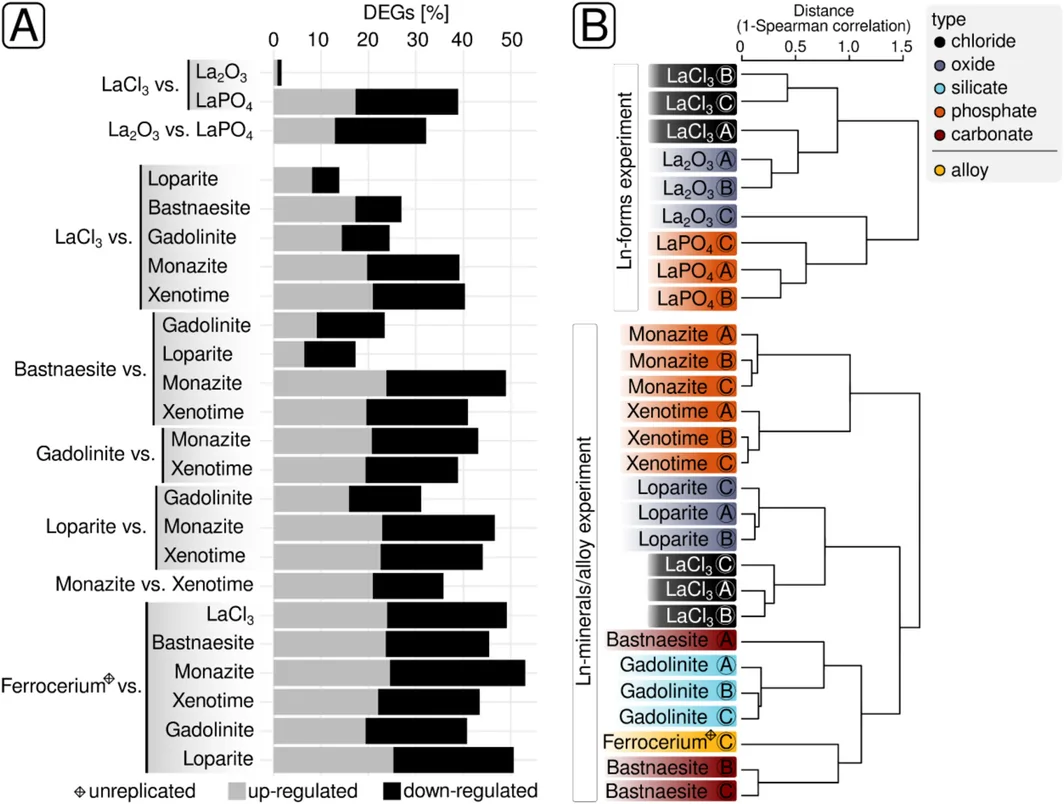
Overview of RNAseq data sets. Statistics regarding up‐ and downregulated genes from differential gene expression analysis are given (A). Data sets from selected samples were clustered based on Spearman distances (B). Each condition was represented by triplicates, except ferrocerium (marked ⊠), due to the high iron load interfering with RNA extraction. Percentages are calculated based on the total number of genes in the genome of the *Beijerinckiaceae* bacterium RH AL1. DEGs = differentially expressed genes.

We used transcriptome‐wide gene expression analysis to examine the effects of the Ln sources on overall metabolism. Differential gene expression analysis was performed based on biological triplicates regarding both incubation experiments (Table S14 [cultivation with different La forms], Table S15 [cultivation with different minerals and ferrocerium]). The ferrocerium incubations yielded only one replicate of sufficient quality for downstream processing. The high iron content likely impaired RNA extraction. Supplementation with different forms of La led to differential gene expression of up to 39% (LaCl_3_ vs. LaO_4_P, 1685 DEGs) and 32% (La_2_O_3_ vs. LaO_4_P, 1392 DEGs) of all encoded genes (Figure 4A, Table S16), with substantial overlap of DEGs (*n* = 1301 genes) between the comparisons (Table S17). Transcriptome analysis of samples cultivated with different Ln‐containing minerals revealed that between 14% (LaCl_3_ vs. loparite, 599 DEGs) and 49% (bastnaesite vs. monazite, 2122 DEGs) of the encoded genes were differentially expressed. Clustering datasets using Spearman distances calculated from scaled CPM (counts per million) values revealed a distinct grouping of Ln phosphates in both incubation experiments (Figure 4B).

### 3.5 Gene Expression Dynamics of the Lanthanome

**FIGURE 5:**
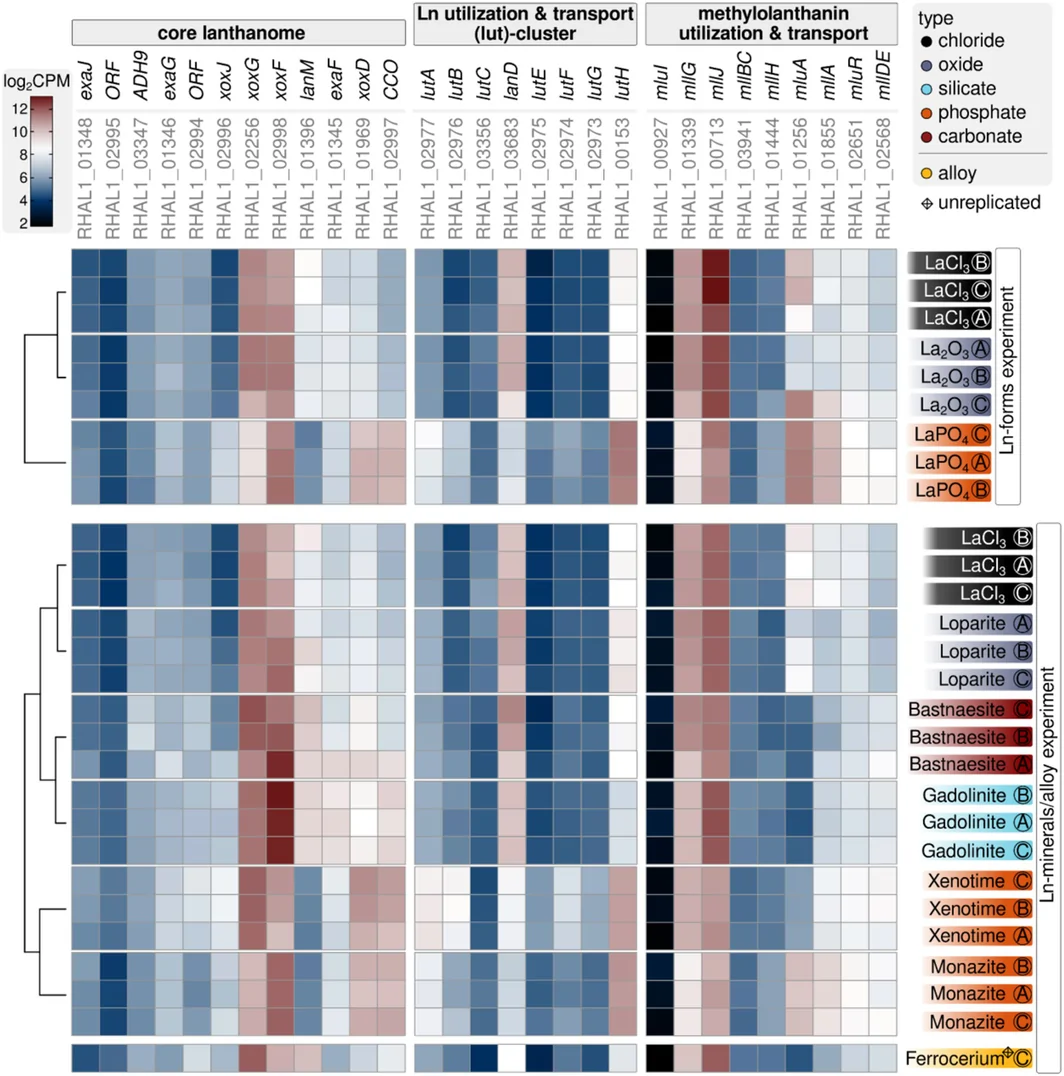
Lanthanome‐related gene expression dynamics. The focus was on core lanthanome genes, genes directly linked to Ln‐driven methylotrophy/alcohol oxidation, as well as gene homologues of the *lut (lanthanide* utilisation and transport) and *mll/mlu* (methylolanthanin/methylolanthanin uptake) clusters. Gene expression is given in log_2_CPM (log_2_ counts per million). The dendrogram illustrates the similarity of the data sets. The single ferrocerium dataset (marked ⊠) was not included in the dendrogram. ORF = open reading frame, CCO = cytochrome C oxidase, ADH9 = gene encoding an Ln‐dependent alcohol dehydrogenase that belongs to subclade 9 within the PQQ ADH family.

A closer look at lanthanome genes (Figure 5, Figure S10, Table S18) revealed that especially Ln phosphates (LaPO_4_, monazite, xenotime) caused notable differences in gene expression (Figure 5). These were particularly evident when looking at *lanM* (RHAL1_01396, encoding the periplasmic Ln‐binding protein lanmodulin), *lanD* (RHAL1_03683, coding for landiscernin, a protein supposed to interact with LanM), *lutH* (RHAL1_00153), and *xoxJ* (RHAL1_02996). Compared to LaCl_3_, La_2_O_3_, and non‐phosphate Ln minerals, *lanM* and *lanD* were downregulated (log_2_FC *lanM* between −2.05 and −3.75, log_2_FC *lanD* between −1.49 and −2.83) (Table S19). The *lutH* gene (coding for a TonB‐dependent receptor involved in periplasmic Ln uptake) was upregulated, reaching log_2_CPM values between 10.32 and 11.19 in samples originating from incubations with Ln phosphates (Figure 5, Figure S7). The *xoxJ* gene (RHAL1_02996, XoxJ is assumed to be involved in XoxF activation) showed broad expression ranging from 4.41–8.22 log_2_CPM (Table S18) and was strongly upregulated when comparing phosphates to LaCl_3_ (log_2_FC 2.91–3.58) (Figure S10), (Table S19).

The expression of *xoxF* and *xoxG*, coding for the Ln‐dependent methanol dehydrogenase and its physiological electron acceptor, a c_L_‐type cytochrome, central to Ln utilisation in AL1, was overall high (*xoxF* log_2_CPM 9.72–13.03, *xoxG* 9.11–11.88). This was independent of the Ln source. When Ln phosphates were omitted, multiple genes associated with the *lut*‐cluster were found to be nonresponsive to differences in lanthanide supplementation. This also included *lutAEF*, which encode the periplasmic binding protein (*lutA*), ATP‐binding cassette (*lutE*), and transmembrane component (*lutF*) of an ABC transporter presumably involved in Ln import from the peri‐ into the cytoplasm. Gene homologues of the *mll−*/*mlu‐cluster* partially responded to different Ln sources. Compared to LaCl_3_, *mluA* (RHAL1_01256, encoding a TonB‐dependent receptor) was downregulated when AL1 was grown with bastnaesite, and gadolinite (log_2_FC between −3.66 and −4.01). We noted differences in gene expression responses, dependent on the different Ln phosphates.

### 3.6 Identification of Clusters of Correlated Genes

**FIGURE 6:**
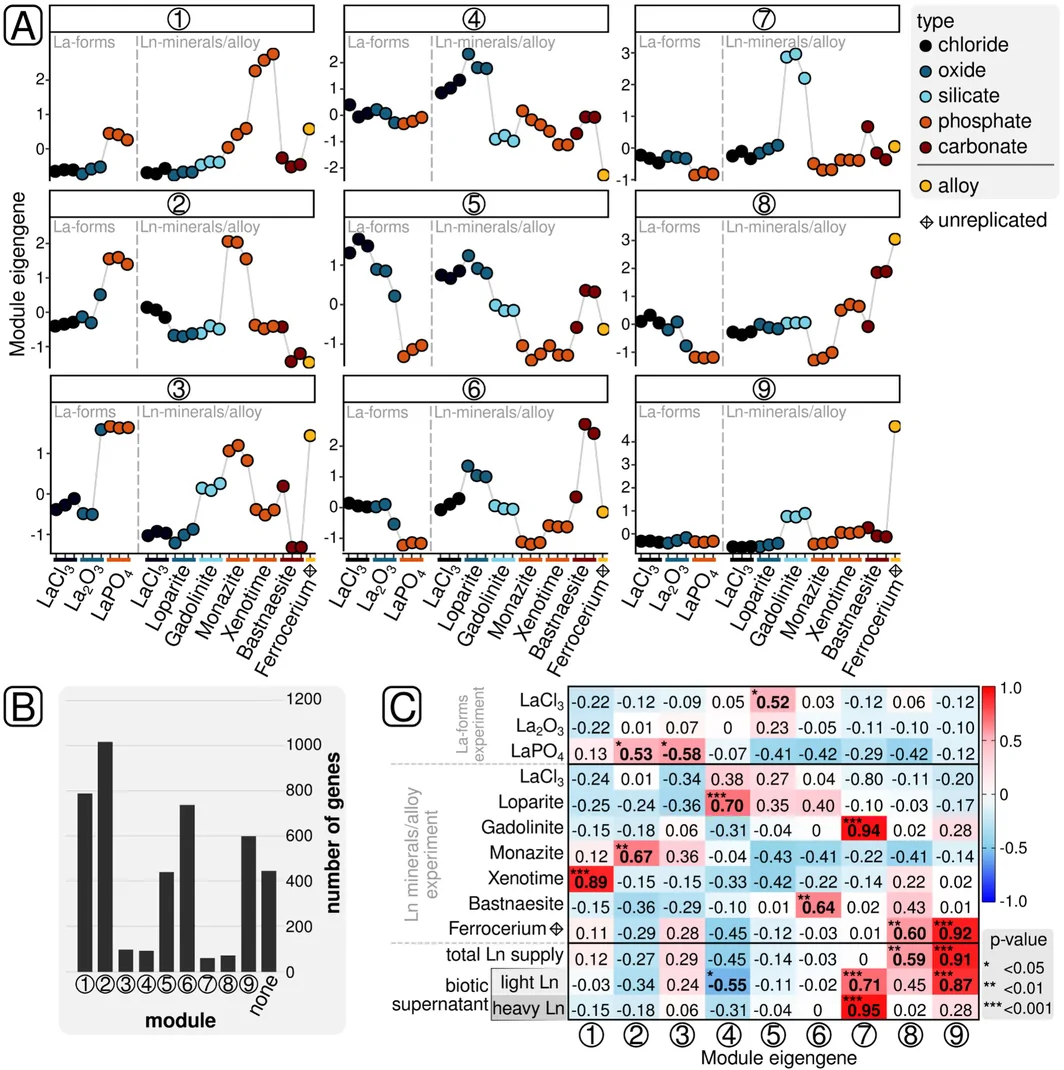
Gene correlation network analysis. Eigengene expression profiles have been deduced from modules obtained via weighted gene co‐expression network analysis (WGCNA). WGCNA led to the identification of nine modules, and the expression profiles of respective module eigengenes are shown (A). Sample colours reflect the respective type of Ln source, which was supplied for cultivation. Ferrocerium is marked (⊠), as the underlying RNAseq data were not replicated. Modules comprised between 60 and 1015 genes (B). Pearson correlations between module eigengenes and selected traits (Ln sources, total Ln supply, and Ln content in biotic supernatant) have been calculated (C). Different significance levels are highlighted.

Network analysis based on weighted gene co‐expression identified 9 modules comprising between 60 and 1015 genes. Gene expression data were summarized module‐wise based on eigengenes (ME‐1 through ME‐9) (Figure 6A+B, Table S20). The response to different Ln phosphates varied, particularly among genes assigned to modules 1–3 and 8. Correlation analysis showed a positive link between individual eigengenes (ME‐1 to ME‐3) and different Ln phosphates (Figure 6C). We also observed a positive correlation between ME‐7 and the silicate gadolinite. Selected modules were examined, focusing on genes with high module membership (MM, the correlation between individual module genes and the respective eigengene) (Figure S11) and strong correlations with Ln sources (Figure S12). We were especially interested in genes putatively associated with Ln mobilisation, uptake, storage, and discrimination.

To test whether these gene expression patterns are a consequence of the Ln sources themselves and not due to exemplary covariates like lanthanide availability, iron content, or observed differences in growth (Figure 2B+C) described above, we performed a sequential PERMANOVA on the transcriptome dataset (Table S7). The identity of the Ln source explained substantial additional variance in gene expression beyond these three covariates combined (pseudo‐F = 19.6, *p* = 0.0001). This indicates that factors specific to each Ln source are a genuine driver of the gene expression patterns described below.

**FIGURE 7:**
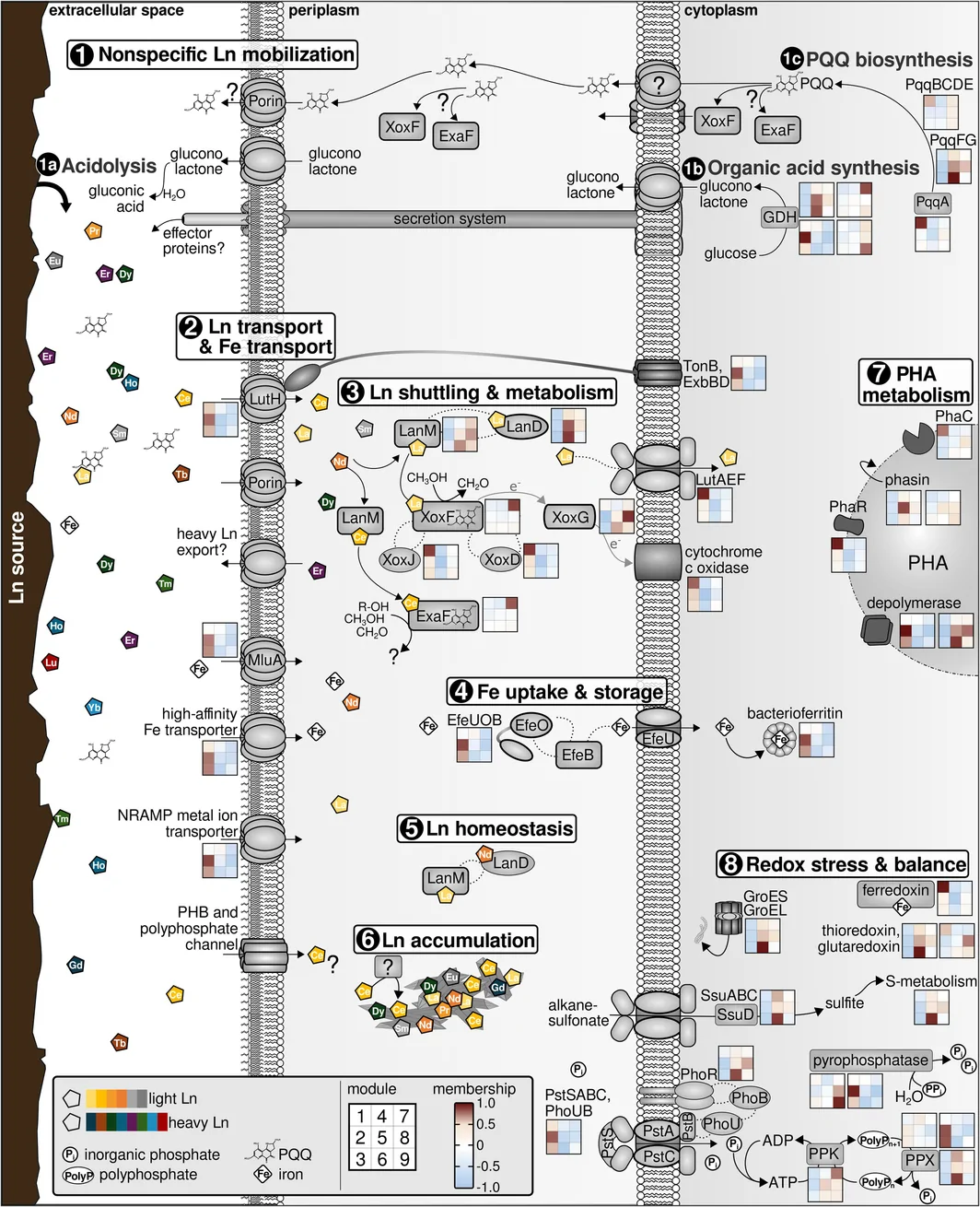
Physiological response to changes in Ln supplementation based on module membership. The individual heatmaps indicate the module membership of the genes encoding the shown proteins and groups of proteins, which were selected based on module memberships and correlations with Ln sources. The focus was on proteins assumed to be involved in Ln mobilization, uptake, sorting, and Ln‐related physiology. Parts of the lanthanome are visualised, including Ln‐dependent ADHs as key enzymes for methanol oxidation and Ln‐binding proteins involved in Ln shuttling and potentially homeostasis. In addition, module memberships of genes encoding proteins involved in polyhydroxyalkanoate (PHA) biosynthesis and degradation are shown, as well as cellular mechanisms involved in redox stress and balance. GDH = glucose dehydrogenase, PPK = polyphosphate kinase, PPX = exopolyphosphatase.

Module‐wise gene expression patterns were mapped onto Ln mobilisation, uptake, storage, and overall metabolism (Figure 7). Module 1 contained several genes associated with PQQ biosynthesis and the lanthanome. We observed particularly high MM for three *pqqA* copies (RHAL1_01618, RHAL1_02672, RHAL1_02989, coding for PqqA, the precursor peptide for PQQ synthesis) and *lut*‐cluster genes *lutABEFG* (RHAL1_02977—RHAL1_02973) (MM between 0.857 and 0.972, *p* < 0.001). The genes *lutH* and *mluA* (RHAL1_01256), encoding TonB‐dependent receptors, were grouped into module 2 (MM 0.765 and 0.719, *p* < 0.001). A closer look at this module revealed various genes associated with iron homeostasis, including uptake (RHAL1_p00067, high‐affinity iron transporter; RHAL1_03444‐RHAL1_03446, siderophore‐independent Fe uptake system EfeUOB) and storage (RHAL1_00987, bacterioferritin) (MM between 0.741 and 0.901, *p* < 0.001). Module 2 also contained genes *pstSABC* and *phoUB* (RHAL1_00909—RHAL1_00914) associated with phosphate transport (MM between 0.708 and 0.878, *p* < 0.001). The genes *xoxF* (RHAL1_02998) and *exaF* (RHAL1_01345) showed distinct expression profiles compared to other lanthanome‐associated genes and were grouped in module 7 (MM 0.861 and 0.811, *p* < 0.001). Module 6 comprised multiple genes associated with redox stress and balance, including *ssuABCD* (RHAL1_02408—RHAL1_02411, MM between 0.826 and 0.917, *p* < 0.001) linked to alkanesulfonate uptake. Several chaperone‐encoding genes, including *groEL* and *groES*, showed high membership in module 6, as did genes encoding glutaredoxin and thioredoxin. Both *lanM* (RHAL1_01396, MM = 0.696 with *p* < 0.001) and *lanD* (RHAL1_03683, MM = 0.871 with *p* < 0.001) were part of module 6, but MM was lower for *lanM*.

The genome of AL1 contains four glucose dehydrogenase genes (RHAL1_00125, RHAL1_01212, RHAL1_02698, and RHAL1_3913), which are linked to gluconolactone (a potential metal chelator) formation. All four of these genes clustered into different modules (modules 6 through 9, MM between 0.765 and 0.947, *p* < 0.001) (Table S20).

## 4 Discussion

Our findings suggest that its metabolic flexibility equips *Beijerinckiaceae* bacterium RH AL1 to mobilise, selectively take up, and store utilisable Lns from diverse sources that differ in type and Ln content. The accessibility and availability of light, utilisable Lns, but not the overall Ln content, controlled Ln‐dependent, methylotrophic growth. Ln‐dependent PQQ ADHs are, despite intense research, still the only known class of Ln‐dependent enzymes (Keltjens et al. 2014; Daumann et al. 2022), though recent work suggests a broader role for Ln‐ and PQQ‐dependent enzymes in carbon substrate transformations (Voutsinos et al. 2026). Ln‐utilising microbes feature a preference for light Lns (Daumann 2019), which is rooted in PQQ ADHs being paired with c_L_‐type cytochromes as physiological electron acceptors. The incorporation of heavy Lns as cofactors in Ln‐dependent PQQ ADH must be avoided by a discrimination mechanism during Ln handling. It was proposed that the reduction potential of XoxG, the physiological electron acceptor of XoxF, limits the range of utilisable Lns (Featherston et al. 2019). Besides, heavy Lns can impair PQQ ADH activity through differences in Lewis acidity and size, the latter potentially perturbing the active site (Lumpe et al. 2018; Singer et al. 2023).

Acidolysis, the dissolution of solids by decreasing the pH, forms the backbone of Ln mobilisation in AL1. Based on our gene expression data, it is presumably driven by the release of organic acids such as gluconic acid (the hydrolysis product of gluconolactone (GL)), which is well known among phosphate‐solubilising bacteria studied for metal‐ and Ln‐leaching (Shin et al. 2015; Corbett et al. 2017; Fathollahzadeh et al. 2019). The genome of strain AL1 encodes multiple glucose dehydrogenases, potentially involved in GL formation. Gene correlation network analysis suggested fine‐tuned expression depending on the Ln source. Synthesis and release of GL by strain AL1 remain to be shown.

Acidolysis alone exerts little selectivity, but pH‐dependent speciation can cause modest fractionation during dissolution itself, especially if elements are hosted in surface complexation sites. The analysis of (a)biotic supernatants, periplasmic deposits, and the calculation of complementary enrichment factors indicated the preferential depletion of light Lns, suggesting Ln discrimination during uptake. Organic acids such as citric acid, oxalic acid, and gluconic acid are potent metal chelators with Ln‐binding preferences based on differences in Lewis acidity, ionic radii, and coordination geometry (Prodius et al. 2020; Meng et al. 2023; Zenker et al. 2025). Besides its role as a cofactor in PQQ ADHs, PQQ was shown, outside of a protein context, to form complexes with preferentially light Lns (Lumpe and Daumann 2019; Lumpe et al. 2020). Differential expression of AL1's *pqqA* genes, encoding the PQQ precursor peptide, suggests a role for PQQ in Ln mobilisation and uptake, especially with respect to Ln phosphates. The availability and interplay between different chelators can aid AL1 in discriminating Lns during mobilisation and uptake.

The identification of the *lut‐* and *mll*−/*mlu*‐clusters (Roszczenko‐Jasińska et al. 2020; Zytnick et al. 2024) provided evidence that Ln uptake by mesophilic bacteria is metallophore‐based, similar to iron and copper uptake, and centred around TonB‐dependent receptors and ABC transporters. Methylolanthanin was the first proposed Ln‐binding metallophore (Zytnick et al. 2024; Gutenthaler‐Tietze et al. 2026), but the binding preferences of methylolanthanin regarding Lns are inconclusive (Gutenthaler‐Tietze et al. 2026). The need for metal‐binding metallophores depends on bioavailability. Organisms such as *Methylacidiphilum fumariolicum* SolV, thriving in acidic systems with high metal loads, have no need for metallophore‐based uptake (Pol et al. 2014). Strain AL1 is probably also less reliant on metal chelators for accessing Lns, as it is a mild acidophile (Wegner et al. 2020; Gorniak et al. 2023).

Gene homologues of the *lut*‐ and *mll*−/*mlu*‐clusters have been identified in AL1, but the observed amino acid sequence identities are partially rather low (Wegner et al. 2020; Gorniak et al. 2023). This dissimilarity was reflected in the nonresponsiveness of some of these genes to changes in Ln supplementation. Notable exceptions were *lutH* and *mluA*, which encode TonB‐dependent receptors within the *lut*‐ and *mll*−/*mlu*‐clusters. The substrates for periplasmic uptake by these receptors are unknown in any organism. Gene expression dynamics and network analysis indicated that LutH may be linked to Ln sensing, while MluA appears to bridge Ln and iron metabolism via EfeUOB and bacterioferritin. EfeUOB is a siderophore‐independent iron uptake system (Cao et al. 2007; Miethke et al. 2013). EfeO was found to bind Sm^3+^ in a recent study (Nakatsuji et al. 2022), and *efeUOB* were differentially expressed in strain AL1 when swapping LaCl_3_ for an equimolar Ln cocktail (Gorniak et al. 2023). Crosstalk between Ln and iron metabolism was suggested for *Pseudomonas alloputida* KT2440 based on overall metal availability (Wehrmann et al. 2019) and supported by observed effects of iron levels on the concentrations of methylolanthanin and cell‐associated Nd in *M. extorquens* AM1 cultivations (Gutenthaler‐Tietze et al. 2026).

We previously proposed that periplasmic Ln deposition is contributing to Ln homeostasis (Wegner et al. 2021; Gorniak et al. 2023). Strain AL1 enriched light, utilisable Ln as mineral‐like periplasmic deposits from all tested sources, despite in part poor solubilities, an overall low Ln content, and low proportions of utilisable Lns. The underrepresentation of heavy Lns in the mineral‐like deposits, together with their overrepresentation in spent medium, supports that Ln discrimination occurs during uptake. The low proportions of heavy Lns taken up seem to be at least partially diverted into the periplasmic deposits. This likely limits their availability in the cytoplasm and the risk of accidentally incorporating them into PQQ ADH undergoing maturation.

As shown before (Wegner et al. 2021; Gorniak et al. 2023), periplasmic accumulation occurred at the cell poles, in close proximity to PHB granules. Correlating gene expression patterns of selected lanthanome‐ and PHA‐related genes supports a link between PHA and Ln metabolism, but this warrants further investigation. Channels composed of complexed short‐chain PHB (polyhydroxybutyrate, a common form of PHA for carbon storage) and polyphosphate represent one important uptake route for calcium (Reusch and Sadoff 1988; Reusch 1992, 2000; Reusch et al. 1995). We previously proposed (Gorniak et al. 2023) an involvement of such channels in Ln uptake, which would explain the localisation of the periplasmic deposits.

Recent work described an intriguing, siphon‐like mechanism for sorting Lns, built around lanmodulin (LanM) and landiscerin (LanD) in *M. extorquens* AM1 (Larrinaga et al. 2024). Heavy Lns are prevented from cytoplasmic uptake and downstream incorporation into PQQ ADHs by transferring them to LanM through LanD. The Ln binding preferences of LanM and LanD match, and LanD is equipped with a novel Ln binding motif, distinct from the heavily studied EF‐hand motif of LanM. We previously showed that *lanM* and *lanD* homologues in AL1 are upregulated when the strain is grown with a mixture of Lns rather than La supporting their involvement in periplasmic Ln sorting (Gorniak et al. 2023). Here, we observed downregulation of *lanM* and *lanD* when AL1 was grown with chemically stable Ln phosphates, including xenotime, which has an elevated load of heavy Lns, likely limiting overall Ln influx. LanM and LanD arguably contribute to the periplasmic Ln balance in strain AL1, but their roles in Ln discrimination remain open questions. The encoded LanD homologue in AL1 differs at the amino acid level with respect to the predicted Ln‐binding site. The proposed roles for LanM and LanD and other mentioned proteins in strain AL1 are based on (co‐)expression patterns. Genetic tools for a more direct functional validation are not yet available.

The assembled gene expression data provide further evidence that Lns reach into many aspects of cellular metabolism. In organisms featuring the Ln switch, gene expression dynamics tend to be limited to the lanthanome and methylotrophy‐related genes (Gu et al. 2016; Masuda et al. 2018; Good et al. 2019; Ochsner et al. 2019; Gorniak et al. 2024). The Ln switch is rather the exception, and organisms without it are more common in the environment (Keltjens et al. 2014; Wilson et al. 2014; Butterfield et al. 2016; Chistoserdova and Kalyuzhnaya 2018). We only start to understand the ecological relevance of the lanthanome (Krause et al. 2017; Voutsinos et al. 2025). We showed that AL1 gene expression was primarily affected by the type of Ln source. A high number of differentially expressed genes indicated a complex metabolic response to the various Ln sources. The mineral type represented the predominant factor driving gene expression changes. Many of the genes we found to be differentially expressed were previously identified by us in our work with Ln chloride salts (Gorniak et al. 2023). These included, among others, genes associated with chemotaxis and motility, polyhydroxyalkanoates, and sulfur metabolism. Natural Ln sources also differed in matrix elements that partially contributed to gene expression changes independent from Lns. Gadolinite and ferrocerium were comparatively iron‐rich, and iron availability is a known regulator of TonB‐dependent receptor expression; module 2, which contains the TonB receptors *lutH* and *mluA*, also includes iron‐uptake and‐storage genes (EfeUOB, bacterioferritin). Monazite and xenotime released phosphate, which was consistent with the assignment of the phosphate transport genes *pstSABC* and *phoUB* to module 2, which shows increased expression in the respective cultures. We cannot fully distinguish these matrix‐element effects from Ln‐specific responses. Our core lanthanome‐associated findings are additionally supported by our past work with soluble Ln chloride salts (Wegner et al. 2021; Gorniak et al. 2023). Our findings presented here and previously underscore the need to use more nature‐inspired Ln sources when studying microbial Ln utilisation. The use of synthetic minerals would facilitate studying relevant aspects such as grain size and surface area in the context of Ln mobilisation and uptake.

Taken together, we suggest that Ln discrimination in *Beijerinckiaceae* bacterium RH AL1 occurs through a multilayered process. Acidolysis provides access to mineral‐bound lanthanides, but confers limited selectivity. Selectivity only arises through the interplay of chelation, different uptake mechanisms, and periplasmic storage, which facilitate the preferential enrichment of utilisable light Lns while heavy Lns are diverted. Ln discrimination in AL1 is sequential with potential implications for bioinspired Ln recovery strategies.

## Author Contributions

**Linda Gorniak:** conceptualization, methodology, data curation, formal analysis, investigation, visualization, writing – original draft, writing – review and editing. **Sophie M. Gutenthaler‐Tietze:** methodology, data curation, formal analysis, investigation. **Alina Lobe:** investigation, formal analysis, data curation. **Lena J. Daumann:** writing – review and editing, resources, methodology. **Robin Steudtner:** methodology, investigation, resources, formal analysis. **Thorsten Schäfer:** resources, writing – review and editing. **Frank Steiniger:** investigation, methodology, data curation, formal analysis. **Martin Westermann:** writing – review and editing, investigation, methodology, formal analysis, data curation. **Kirsten Küsel:** writing – review and editing, resources. **Carl‐Eric Wegner:** conceptualization, methodology, data curation, supervision, project administration, funding acquisition, writing – original draft, writing – review and editing, resources, investigation, formal analysis.

## Funding

This work was supported by the Deutsche Forschungsgemeinschaft, 467713456, 239748522; as well as the European Commission, “Lanthanophore”.

## Conflicts of Interest

The authors declare no conflicts of interest.

## Supporting information


**Figure S1:** Growth curves of *Beijerinckiaceae* bacterium RH AL1 based on repeated incubations with selected Ln sources. Biomass samples for transmission electron microscopy (timepoints highlighted by grey boxes) were collected at matching optical densities to those of the biomass samples taken for RNAseq (Figure 2).
**Figure S2:** Pseudocount distributions for the obtained RNAseq data sets. Shown distributions refer to data sets derived from incubations with different La compounds (upper panel) and the different Ln‐containing minerals and the alloy ferrocerium (lower panel). Pseudocounts were calculated for each gene according to the formula: log_2_([count of mapped reads] + 1). Ferrocerium is marked (⊠), as the underlying RNAseq data were not replicated.
**Figure S3:** Biological coefficients of variation obtained from the different RNAseq data sets. The two plots refer to data sets derived from incubations with different La compounds (upper panel) and the different Ln‐containing minerals and the alloy ferrocerium (lower panel). log_2_CPM = log_2_ counts per million.
**Figure S4:** The distribution of *p*‐values obtained from differential gene expression analysis. Calculations are according to Robinson and Smyth (20) and have been carried out using *edgeR* (14). Ferrocerium is marked (⊠), as the underlying RNAseq data were not replicated.
**Figure S5:** Hierarchical clustering of filtered (log_2_CPM > 4) and scaled gene expression data that were included in weighted gene co‐expression network analysis. *LaCl_3_ refers to samples from the Ln mineral/alloy growth experiment. Ferrocerium is marked (⊠), as the underlying RNAseq data were not replicated.
**Figure S6:** Parameter selection for WGCNA (weighted gene correlation network analysis). Shown are the scale‐free topology model (A) and mean connectivity (B) across selected soft‐threshold powers for filtered and normalized gene expression data from samples grown with the investigated Ln sources.
**Figure S7:** Cluster dendrogram obtained from weighted gene co‐expression network analysis. Nine modules were identified using a soft power of 15 for subsequent calculation of a topological overlap matrix, with a minimum block size of 30 and a merge cut height of 0.30.
**Figure S8:** Elemental analysis of periplasmic, mineral‐like depositions in cultures grown with Ln‐containing phosphate minerals, based on EDX.
**Figure S9:** Elemental analysis of periplasmic, mineral‐like depositions in cultures grown with Ln‐containing minerals bastnaesite, loparite, and gadolinite, based on EDX.
**Figure S10:** Differential gene expression of lanthanome‐associated genes based on log_2_FC values, including genes associated with the core lanthanome (black), the lanthanide utilisation and transport (*lut*‐cluster) gene cluster (dark grey), and homologues of the methylolanthanin utilisation (*mll*−/*mlu*‐cluster) gene cluster (light grey). Ferrocerium is marked (⊠), as the underlying RNAseq data were not replicated.
**Figure S11:** Scatter plots of gene significances for different Ln phosphates and module memberships for genes grouped in selected modules. Colours and shapes highlight associated gene functions.
**Figure S12:** Scatter plots of gene significances for selected traits (Ln sources [ferrocerium, gadolinite], total Ln supply, heavy Ln content in biotic supernatant samples) and module memberships for genes grouped in modules 7–9. Colours and shapes highlight associated gene functions. Ferrocerium is marked (⊠), as the underlying RNAseq data were not replicated.
**Table S1:** Members of the rare earth elements and the lanthanide series.
**Table S2:** Acid digestion of ground Ln minerals and ferrocerium in preparation for analytical investigation via ICP‐MS was performed using an Anton Paar Microwave 5000. To complex excess HF, a complexation run with boric acid (600 μL boric acid per 100 μL HF) was performed. Following acid digestion, samples were adjusted with water (ultrapure, type 1) to 50 mL volumes. Measurements were performed in sample triplicates and technical triplicates.
**Table S3:** Overview about the experimental setups including Ln sources, added Ln concentrations and the number of replicates.
**Table S4:** Ln concentrations and quantities in the used MM2 medium.
**Table S5:** Enrichment factors (EF) for light, utilisable, heavy Lns regarding lanthanide mobilisation (source → supernatant, abiotic and biotic) and uptake/storage (supernatant → deposit). The procedure is outlined in the supplementary material. EFs highlighted in orange were calculated based on lanthanide concentrations in (a)biotic supernatants that were at or below the limit of quantitation (LOQ) of the respective ICP‐MS measurements. We substituted these values with LOQ/2. The resulting EFs indicate the direction of enrichment/depletion. They are not quantitative magnitudes. The underlying Ln proportions (light, utilisable, heavy) of the source, abiotic (pH 3) supernatant, biotic supernatant, and periplasmic deposit for each source, from which the Efs were calculated are given as well. These values are rooted in Tables S4, S11, S13. nc = not calculated.
**Table S6:** Element‐specific lanthanide enrichment factors (EFs) for lanthanide mobilisation (source → supernatant, abiotic and biotic) and uptake/storage (supernatant → deposit). The procedure is outlined in the supplementary material. EFs highlighted in orange were calculated based on lanthanide concentrations in (a)biotic supernatants that were at or below the limit of quantitation (LOQ) of the respective ICP‐MS measurements. We substituted these values with LOQ/2. The resulting EFs indicate the direction of enrichment/depletion. They are not quantitative magnitudes. The bottom four tables below the EF tables indicate the underlying Ln proportions (light, utilisable, heavy) of the source, abiotic (pH 3) supernatant, biotic supernatant, and periplasmic deposit for each source, from which the EFs were calculated. These values are rooted in Tables S4, S11, S13. nc = not calculated.
**Table S7:** Permutational multivariate analysis of variance (PERMANOVA). Sequential PERMANOVA partitioning of whole‐transcriptome variance (Euclidean distances) among abiotically dissolved Ln concentration [1], iron content of the Ln sources [2], growth yield (carrying capacity) [3], and the identity of the Ln source [4]. Df = degrees of freedom, SumOfSqs = sum of squares, R^2^ = proportion of variance explained, F = pseudo‐F ratio (mean square of the term/mean square of the residual), Pr(>F) = permutation‐based *p*‐value (9999 permutations).
**Table S8:** Analytical investigation of Ln mineral compositions via energy‐dispersive X‐ray spectroscopy (EDX). Five replicates per sample were measured using a Helios Nanolab G3 Dual Beam UC (FEI, part of ThermoFisher Scientific, Darmstadt, Germany) with an X‐Max 80 SDD detector (Oxford Instruments GmbH, Wiesbaden, Germany).
**Table S9:** Analytical investigation of Ln mineral compositions via ICP‐MS. Measurements were performed in sample triplicates and technical triplicates using an iCAP RQ ICP‐MS device (Thermo Fisher Scientific, Frankfurt, Germany). LOD = limit of detection.
**Table S10:** Growth metrics for each tested Ln source based on logistic growth model parameters from growthcurver. k = carrying capacity, k_se/k_p = standard error/p‐value for k, n0 = initial population size, n0_se/n0_p = standard error/*p*‐value for n0, *r* = growth rate, r_se/r_p = standard error/p‐value for r, sigma = residual standard error of the fit, df = degrees of freedom, t_mid = time to reach half of carrying capacity, t_gen = generation (doubling) time, auc_l/auc_e = area under the empirical/fitted logistic curve.
**Table S11:** Supernatant analytics, determined via ICP‐MS. Measurements of selected elements (Ln elements, Be, Ca, Fe, Na, Nb, P, Si, Sr., Ti, Y) were performed with biological triplicates and technical triplicates. Controls without Ln supplementation MM2 medium with methanol (0.5% v/v, 123 mM), and controls with methanol (0.5% v/v, 123 mM) but no Ln addition were only measured in technical triplicates. SD = standard deviation.
**Table S12:** Extent of abiotic lanthanide dissolution at pH 3.
**Table S13:** Investigation of periplasmic Ln accumulations via EDX. The deconvolution analysis of EDX spectra returned the following results. Ln accumulations were analysed from strain AL1 grown with the Ln minerals apatite, bastnaesite, gadolinite, loparite, monazite, and xenotime.
**Table S14:** Overview of gene expression data (log_2_ counts per million, log_2_CPM) of strain AL1 in response to different La forms (chloride, oxide, or phosphate, 1 μM). Data were obtained from biological triplicates.
**Table S15:** Overview of gene expression data (log_2_ counts per million) of strain AL1 in response to different Ln minerals (bastnaesite, gadolinite, loparite, monazite, xenotime), ferrocerium, or LaCl3 (1 μM). Data were obtained from biological triplicates except ferrocerium.
**Table S16:** Overview of the numbers of differentially expressed genes in both experiments. Differentially expressed genes (DEGs) were defined based on a false discovery rate (FDR) < 0.05, log_2_ fold changes (log_2_FCs) > 0.58 or < (−0.58), and log_2_ counts per million (log_2_CPM) > 4. DEGs [%] describes the calculated percentages based on the total number of 4323 encoded in the genome of AL1.
**Table S17:** Matrix containing log_2_ fold changes (log_2_FCs) of differentially expressed genes, and the number of comparisons in which differential gene expression (DGE) was observed. Empty slots refer to no differential gene expression in the corresponding comparison(s). Gene annotation data was taken from Wegner et al. 2020.
**Table S18:** Overview of the gene expression of lanthanome(−related) genes based on log_2_CPM values. Genes were grouped as described in the main text. Homologues for the mll−/mlu‐cluster from *M. extorquens* AM1, however, showed low homology on the amino acid sequence level (< 30% sequence identity) Wegner et al. 2020.
**Table S19:** Overview of differential gene expression of lanthanome‐associated genes (log_2_FCs). Genes were grouped as described in the main text. Empty slots refer to no differential gene expression in the corresponding comparison(s).
**Table S20:** Gene clustering based on weighted gene co‐expression network analysis. Gene significance (GS) for selected traits and module membership (MM) are listed with corresponding *p*‐values.

## Data Availability

The data that support the findings of this study are openly available in EBI ArrayExpress at https://www.ebi.ac.uk/biostudies/arrayexpress/studies/E‐MTAB‐15640, reference number E‐MTAB‐15640. Further details regarding data processing are available through GitHub (https://github.com/wegnerce/gorniak_et_al_2026). A reproducible, snakemake‐based (Köster and Rahmann 2012; Mölder et al. 2021) workflow for RNAseq data processing can be retrieved from GitHub (https://github.com/wegnerce/smk_rnaseq, release v0.1).
