# Supplementary material for: Source‐Independent Enrichment of Light Lanthanides: Microbial Mobilization, Selective Uptake, and Intracellular Storage
Source: Environ Microbiol. 2026 Sep 16;28(9):e70413. doi: 10.1111/1462-2920.70413 (PMC13583147; doi:10.1111/1462-2920.70413)
Supplement: Supplementary file 1 [file EMI-28-e70413-s001.pdf]

SUPPLEMENTARY MATERIAL: **Source-independent enrichment of light lanthanides: microbial mobilization, selective uptake, and intracellular storage**

Linda Gorniak[1,2], Sophie M. Gutenthaler-Tietze[3,4], Alina Lobe[4], Lena J. Daumann[3,4], Robin Steudtner[5], Thorsten Schäfer[2], Frank Steiniger[6], Martin Westermann[6], Kirsten Küsel[1,7,8], Carl-Eric Wegner[1,9]

[1]Institute of Biodiversity, Aquatic Geomicrobiology, Friedrich Schiller University, Dornburger Str. 159, 07743 Jena, Germany

[2]Applied Geology, Institute of Geosciences, Friedrich Schiller University, Burgweg 11, 07749 Jena, Germany

[3]Chair of Bioinorganic Chemistry, Heinrich Heine University, Universitätsstr. 1, 40225 Düsseldorf, Germany

[4]Department of Chemistry, Ludwig Maximilian University, 80539 München, Germany

[5]Institute of Resource Ecology, Helmholtz-Zentrum Dresden-Rossendorf e.V, Bautzner Landstraße 400, 01328 Dresden,Germany

[6]University Hospital Jena, Electron Microscopy Center, Ziegelmuehlenweg 1, 07743, Jena, Germany

[7]Cluster of Excellence Balance of the Microverse, Friedrich Schiller University Jena, Jena, Germany

[8]German Center for Integrative Biodiversity Research (iDiv) Halle-Jena-Leipzig, Germany

[9]Chair of Bioinorganic Chemistry, Biomics Group, Heinrich Heine University, Universitätsstr. 1, 40225 Düsseldorf, Germany

[#]corresponding author: Carl-Eric Wegner | carl-eric.wegner@hhu.de

SUPPLEMENTARY MATERIAL

Supplementary Methods. Complementary information about the used materials and applied methods.

We provide further details about sequence data processing, differential gene expression analysis, and weighted gene correlation network analysis through a GitHub repository (https://github.com/wegnerce/gorniak_et_al_2026).

Analytical investigation of the Ln minerals by ICP-MS (inductively coupled plasma mass spectrometry)

Ln-containing minerals were fine-ground using an achat mortar (gadolinite, loparite) or a Vibratory Micro Mill PULVERISETTE 0 (Fritsch, Idar-Oberstein, Germany). The latter was used at an amplitude of ×2.0 for the minerals bastnaesite, apatite (10 minutes), and xenotime (4 minutes). Monazite was milled for 2 minutes at an amplitude of ×1.5. Only the ferrocerium could not be ground due to sparking and was used in pieces of 145.27 ± 2.48 mg. Microwave-assisted acid digestion of Ln compounds and respective blanks was carried out in triplicates using a Multiwave 5000 (Anton Paar GmbH, Graz, Austria), sulphuric acid (98%, Merck KGaA, Darmstadt, Germany), nitric acid (69%, ARISTAR® for trace analysis, VWR International GmbH, Darmstadt, Germany), hydrochloric acid (37%, ARISTAR® for trace analysis, VWR International GmbH, Darmstadt, Germany), and/or hydrofluoric acid (47 - 51%, TraceSELECT™ for trace analysis, Honeywell Deutschland Holding GmbH, Offenbach, Germany) (**Table S2**). Sample volumes were adjusted with water (ultrapure, type 1) to 50 mL, and inductively coupled plasma mass spectrometry was performed at the analytical facility of the Helmholtz Center Dresden-Rossendorf,

Germany, using an iCAP RQ ICP-MS device (Thermo Fisher Scientific, Frankfurt, Germany).

Cultivation

Pre-cultures of *Beijerinckiaceae* bacterium RH AL1 were grown with glucose (278 mM, 0.05% [w/v]) and harvested biomass washed five times with 20 mL MM2 basal medium (1, 2), before being used as inoculum for subsequent incubations with methanol (0.5% [v/v], 123 mM) as the carbon source. For each condition (i.e., different Ln source), incubations were performed in triplicate in acid-washed 250 mL Erlenmeyer flasks sealed with cotton stoppers. Cultivations were carried out at room temperature on a shaker (100 rpm) with a total cultivation volume of 140 mL. Two sets of incubations were done. For the first one, La or Nd (1 µM) were supplied in different forms: as (i) chlorides, (ii) oxides, or (iii) phosphate (La only). After 4 days of cultivation, biomass samples for RNA extraction were collected during late exponential growth (OD_{600nm} between 0.178 ± 0.015 and 0.422 ± 0.093). Due to significantly slower growth, cultures grown with $LaPO_4$ were sampled after 16 days of cultivation, when optical density values (0.386 ± 0.014) were approximately the same as those previously sampled for La chloride and oxide cultures (0.379 ± 0.038 and 0.422 ± 0.093). For the second set of incubations, Ln-containing minerals (apatite, bastnaesite, gadolinite, loparite, monazite, or xenotime) or the alloy ferrocerium served as Ln sources. Milled minerals (5 mg) have been subjected to dry-heat sterilization. Ferrocerium pieces of 145.27 ± 2.48 mg were autoclaved in a 100% nitrogen-flushed serum bottle before using them as an Ln source for cultivation. Biomass samples for RNA extractions were taken at OD_{600nm} values between 0.321 ± 0.019 and 0.415 ± 0.031 ($LaCl_3$, ferrocerium, loparite, gadolinite, bastnaesite,

monazite). Due to delayed growth, samples from xenotime cultures were taken at OD_{600nm} 0.079 ± 0.005. Cells were harvested by centrifugation (10000 × g, 10 min, at room temperature) and stored at −80°C until further processing. Controls with methanol (0.5% [v/v], 123 mM), but without added Lns, were run in parallel to probe for Ln leaching from glassware and glucose carryover from pre-cultures. Additional controls with Ln minerals or ferrocerium, but no methanol, were used to verify that AL1 did not utilize carbon present in the Ln sources.

Cultivations were repeated for all conditions (except for $NdCl_3$ and Nd_2O_3) as described above. Growth patterns matched those of the previous cultivation. Biomass samples for transmission electron microscopy (TEM) and energy-dispersive X-ray spectroscopy (EDX) were collected during the late exponential growth phase, when optical densities were comparable to those at the sampling timepoints for the RNAseq experiment (**Figure S3**). At respective timepoints, optical densities ranged between OD_{600nm} 0.325 ± 0.013 (apatite) and OD_{600nm} 0.586 ± 0.059 (loparite), except for cultures supplied with xenotime (OD_{600nm} 0.075 ± 0.012) or $LaPO_4$ (OD_{600nm} 0.215 ± 0.076). Growth rates were determined with *growthcurver* (v0.3.1) (3).

Calculation of Ln enrichment factors

Enrichment factors (EFs) have been calculated for light (La-Eu), utilizable (La-Nd), and heavy (Gd-Lu) lanthanides (**Table S5**). Element-specific EFs have been calculated as well (**Table S6**). We distinguished two stages regarding Ln enrichment: source → supernatant (mobilization/dissolution), and supernatant → periplasmic deoposits (uptake/storage). For both, we looked at the lanthanide composition of the different pools (source material, (a)biotic supernatants [abiotic refers here to the abiotic pH3 supernatants], periplasmic deposits). Source compositions were taken

from the molar values (**Table S4)**. Supernatant compositions (abiotic pH 3 and biotic) were deduced from the ICP-MS concentrations ($\mu g \times L^{-1}$) (**Table S11**), and converted to molar concentrations using the atomic weight of each element. Concentrations below the limit of quantitation (LOQ) were substituted with LOQ/2 before calculating molar fractions. Periplasmic deposit compositions were taken from the EDX-derived atomic percentages (**Table S13**), which were renormalized to the lanthanide sum. The resulting EFs indicate the direction of enrichment/depletion. They are not quantitative magnitudes.

Sequence data pre-processing

The quality of raw and trimmed sequences was checked with *fastQC* (v0.11.9) (4). Adaptor- and quality-trimming (settings: minlen = 75, qtrim = rl, ktrim = rl, k = 25, mink = 11, trimq = 20) were done with *bbduk* (v38.26) (5) and its included database of common sequence contaminants and adaptors. Reads derived from rRNA and non-coding RNA have been removed from trimmed datasets with *SortMeRNA* (v2.1) (6) and the SILVA (7, 8) and Rfam (9) databases. Reads were mapped onto the reference genome of *Beijerinckiaceae* bacterium RH AL1 (EBI/ENA accession numbers: LR590083, LR699074 [genome and plasmid]) with *bbmap* (v.38.26) (settings: slow, k = 11). The number of mapped reads per feature (e.g. coding genes) was deduced from generated *.bam* files, sorted and indexed with *samtools* (v1.3.1) (10), using *featureCounts,* which is part of the *Subread* package (v1.6.3) (11, 12).

Differential gene expression analysis

Differential gene expression analysis was performed using the R software framework (v.4.2.1) (13) and the packages *edgeR* (v.3.42.4) (14), *limma* (v.3.56.2) (15), *mixOmics* (v.6.24.0) (16), *HTSFilter* (v.1.40.0) (17), *bigPint* (v.1.15.0) (18), as well as their respective dependencies. Pseudo-counts (log_2(counts+1)) (**Figure S4**) were generated to examine read count data through MA (mean of the normalized counts versus the log_2FC [fold change] for all genes tested) plots. Multidimensional scaling plots were prepared with *limma,* and scaled read count data were used to check inter-dataset relationships. Scatterplot matrices were plotted with the *plotSM* function of *bigPint* to assess correlations between intra-sample and inter-sample replicates, respectively. Genes that recruited insufficient numbers of reads were removed before downstream analysis. For filtering, we used the filterByExpr function in *edgeR*. Sample normalization was performed using calculated normalization factors and assessed dispersion. The uncertainty in transcript abundances across replicates was accounted for by calculating the biological coefficient of variation (BCV) (**Figure S5**). We determined p-values deduced from the exact test established by Robinson and Smyth (14) for all carried-out comparisons (**Figure S6**). Putative differentially expressed genes were filtered based on log_2 fold change (log_2FC), FDR-corrected *p*-value, and gene expression in log_2counts per million (cpm). Genes featuring changes in gene expression above |0.58| log_2FC (fold change), gene expression values higher than 4 (log_2CPM, counts per million), and p-values smaller than 0.05 were considered for further analysis.

Permutational multivariate analysis of variance (PERMANOVA)

We tested whether gene expression changes can be linked to covariates such as iron content, Ln availability, and observed differences in growth rather than the

identity of the Ln sources themselves. We performed a sequential permutational multivariate analysis of variance (PERMANOVA) on Euclidean distances calculated from standardized (z-scored) $\log_2$CPM values using the *adonis2* function implemented in *vegan* (v2.7.5) (19).Genes with a mean $\log_2$CPM > 4 across the samples analyzed (n = 3,581) were considered. The analysis included six different Ln sources with matched triplicate transcriptomic data ($LaCl_3$, bastnaesite, gadolinite, loparite, monazite, xenotime; n = 18 samples). Covariates were analyzed sequentially in the following order: abiotic-dissolved lanthanide concentration (**Table S11**), the iron content of the source (**Table S9**), biomass yield (=carrying capacity determined with *growthcurver* (v0.3.1) (3), and finally the identity of the Ln source. This order was chosen to assess the explained shared variance for each of the potentially confounding variables. As a result, we tested whether the identity of the Ln source explained shared variance beyond these covariates. Statistical significance was assessed by 9,999 permutations (**Table S7**).

Supplementary results. Complementary information regarding the obtained results.

Elemental composition of the studied minerals

The composition of the mineral ores was determined by EDX, and allowed the calculation of sum formulas $Ca_{8.70}(P_{0.92}O_4)_6F_{2.20}$ for (fluor)apatite, $(La - Sm)_{0.96}P_{1.04}O_4$ for monazite, and $(Y, Gd, Dy, Er, Yb)_{0.90}P_{0.94}O_4$ for xenotime. These phosphate mineral ores contained between 13.5% ± 0.8% (apatite) and 17.3% ± 1% (monazite) phosphorus. Minor impurities were observed in some of the five replicates per ore, including Si (≤ 0.4%), Ca (≤ 0.15% in monazite ores), Na (≤ 0.15% in apatite ores), or Sr (0.8% ± 0.05% in apatite ores). Detailed descriptions of ore compositions can be found in **Tables S8+S9**. A sum formula of $(La, Ce, Nd, Na, Ca)_{1.68}(Ti, Nb)_{2.08}O_6$ could be calculated for loparite. Silicate mineral gadolinite exhibited the elemental proportions $(Ce, Pr, Nd, Sm, Gd, Dy, Yb, Y)_{1.79}Fe_{0.78}Be_{2.15}Si_{1.89}O_{10}$. The beryllium proportion was assessed using ICP-MS. Bastnaesite represents an Ln-containing fluorocarbonate that often co-occurs with minerals calcite, fluorite, barite, celestite, and dolomite. A carbon content of only 6.10 % (based on elemental analysis) and the presence of Ca (4.73 at.%), Ba (6.76 at.%), S (5.92 at.%), and Sr (4.84 at.%) indicated that the ore was not pure. We have referred to the ore as bastnaesite for simplicity.

Growth of Beijerinckiaceae bacterium RH AL1 with different Ln minerals/alloy

Cultivations were repeated (except for $NdCl_3$ and Nd_2O_3) as described in the materials and methods section in the main text. As previously observed, supplementation of $LaCl_3$ or Ln oxides resulted in only minimal changes in growth patterns (growth rates μ between 0.030 ± 0.001 h^{-1} and 0.037 ± 0.003 h^{-1}) (**Figure**

S3). Growth was notably slower in cultures grown with xenotime (μ = 0.013 ± 0.002 h^{-1}) or $LaPO_4$ (μ = 0.014 ± 0.002 h^{-1}). Fast growth (μ = 0.060 ± 0.004 h^{-1}) and high final optical densities (OD_{600nm} 1.242 ± 0.030) were detected in cultures grown with ferrocerium. Observations were supported by cell counts based on a Thoma counting chamber. While ferrocerium cultures reached a cell concentration of $3.28 \times 10^8 \pm 1.36 \times 10^7$ cells/mL after 73 hours of incubation, concentrations of $1.57 \times 10^8 \pm 1.57 \times 10^7$ cells/mL were determined for $LaCl_3$ cultures after 113 hours of incubation (mid- to late-exponential sampling timepoints).

SUPPLEMENTARY FIGURES

Figure S1. Growth curves of *Beijerinckiaceae* bacterium RH AL1 based on repeated incubations with selected Ln sources. Biomass samples for transmission electron microscopy (timepoints highlighted by grey boxes) were collected at matching optical densities to those of the biomass samples taken for RNAseq (**Figure 2**).

Figure S2. Pseudocount distributions for the obtained RNAseq data sets. Shown distributions refer to data sets derived from incubations with different La compounds (upper panel) and the different Ln-containing minerals and the alloy ferrocerium (lower panel). Pseudocounts were calculated for each gene according to the formula: $\log_2$([count of mapped reads]+1). Ferrocerium is marked (⊠), as the underlying RNAseq data were not replicated.

Figure S3. Biological coefficients of variation obtained from the different RNAseq data sets. The two plots refer to data sets derived from incubations with different La compounds (upper panel) and the different Ln-containing minerals and the alloy ferrocerium (lower panel). $\log_2$CPM = $\log_2$ counts per million.

Figure S4. The distribution of p-values obtained from differential gene expression analysis. Calculations are according to Robinson and Smyth (20) and have been carried out using *edgeR* (14). Ferrocerium is marked (⊠), as the underlying RNAseq data were not replicated.

Figure S5. Hierarchical clustering of filtered ($\log_2$CPM > 4) and scaled gene expression data that were included in weighted gene co-expression network

analysis. *$LaCl_3$ refers to samples from the Ln mineral/alloy growth experiment. Ferrocerium is marked (☒), as the underlying RNAseq data were not replicated.

Figure S6. Parameter selection for WGCNA (weighted gene correlation network analysis). Shown are the scale-free topology model (upper panel) and mean connectivity (lower panel) across selected soft-threshold powers for filtered and normalized gene expression data from samples grown with the investigated Ln sources.

Figure S7. Cluster dendrogram obtained from weighted gene co-expression network analysis. Nine modules were identified using a soft power of 15 for subsequent calculation of a topological overlap matrix, with a minimum block size of 30 and a merge cut height of 0.30.

Figure S8. Elemental analysis of periplasmic, mineral-like depositions in cultures grown with Ln-containing phosphate minerals, based on EDX.

Figure S9. Elemental analysis of periplasmic, mineral-like depositions in cultures grown with Ln-containing minerals bastnaesite, loparite, and gadolinite, based on EDX.

Figure S10. Differential gene expression of lanthanome-associated genes based on log_2FC values, including genes associated with the core lanthanome (black), the lanthanide utilization and transport (*lut*-cluster) gene cluster (dark grey), and homologues of the methylolanthanin utilization (*mll*-/*mlu*-cluster) gene cluster (light

grey). Ferrocerium is marked (⊠), as the underlying RNAseq data were not replicated.

Figure S11. Scatter plots of gene significances for different Ln phosphates and module memberships for genes grouped in selected modules. Colors and shapes highlight associated gene functions.

Figure S12. Scatter plots of gene significances for selected traits (Ln sources [ferrocerium, gadolinite], total Ln supply, heavy Ln content in biotic supernatant samples) and module memberships for genes grouped in modules 7 - 9. Colors and shapes highlight associated gene functions. Ferrocerium is marked (⊠), as the underlying RNAseq data were not replicated.

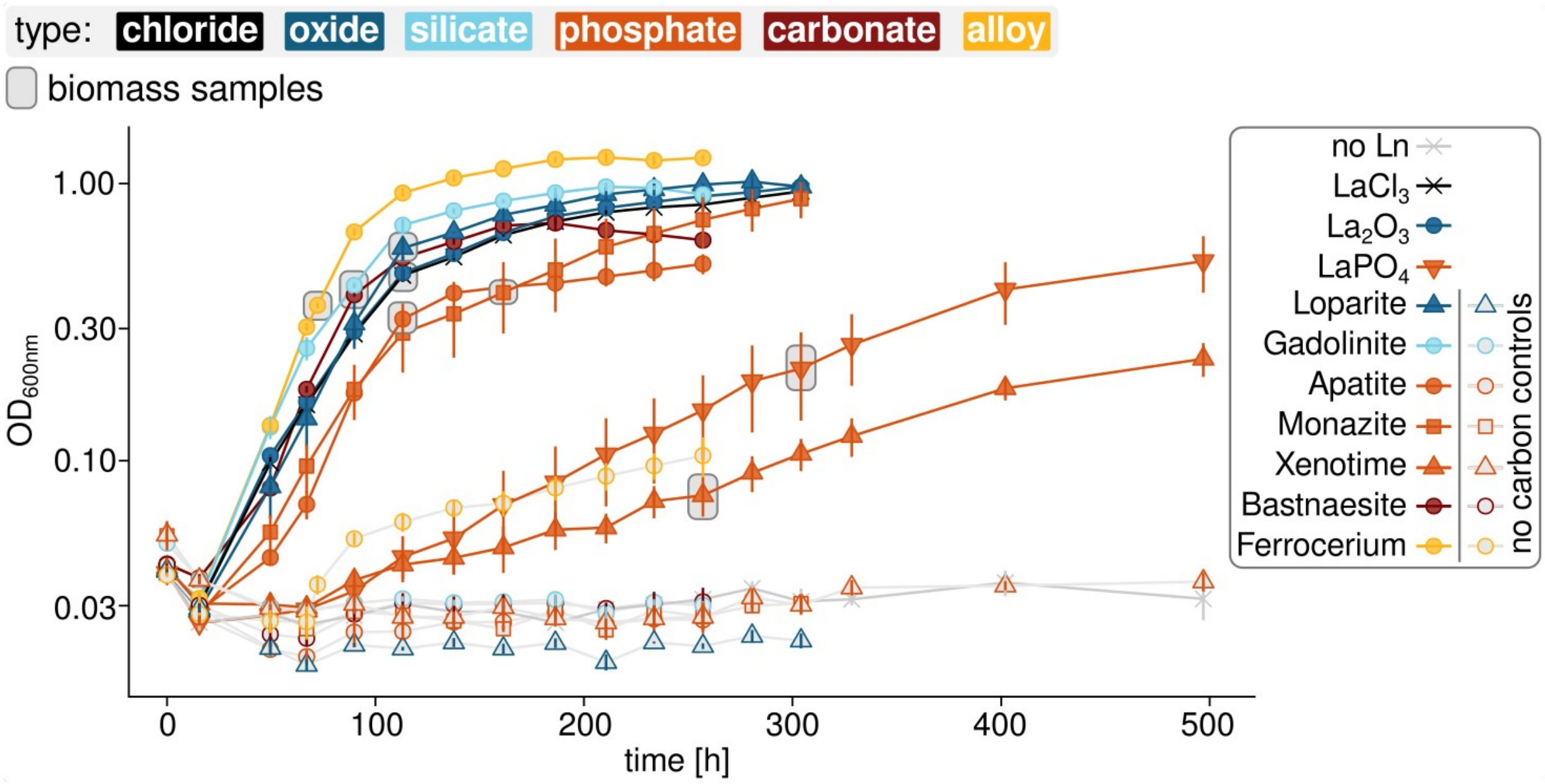
type:
chloride
oxide
silicate
phosphate
carbonate
alloy
biomass samples
OD600nm
1.00
0.30
0.10
0.03
0
100
200
300
400
500
time [h]
no Ln
LaCl3
La2O3
LaPO4
Loparite
Gadolinite
Apatite
Monazite
Xenotime
Bastnaesite
Ferrocerium
no carbon controls

Figure S1.

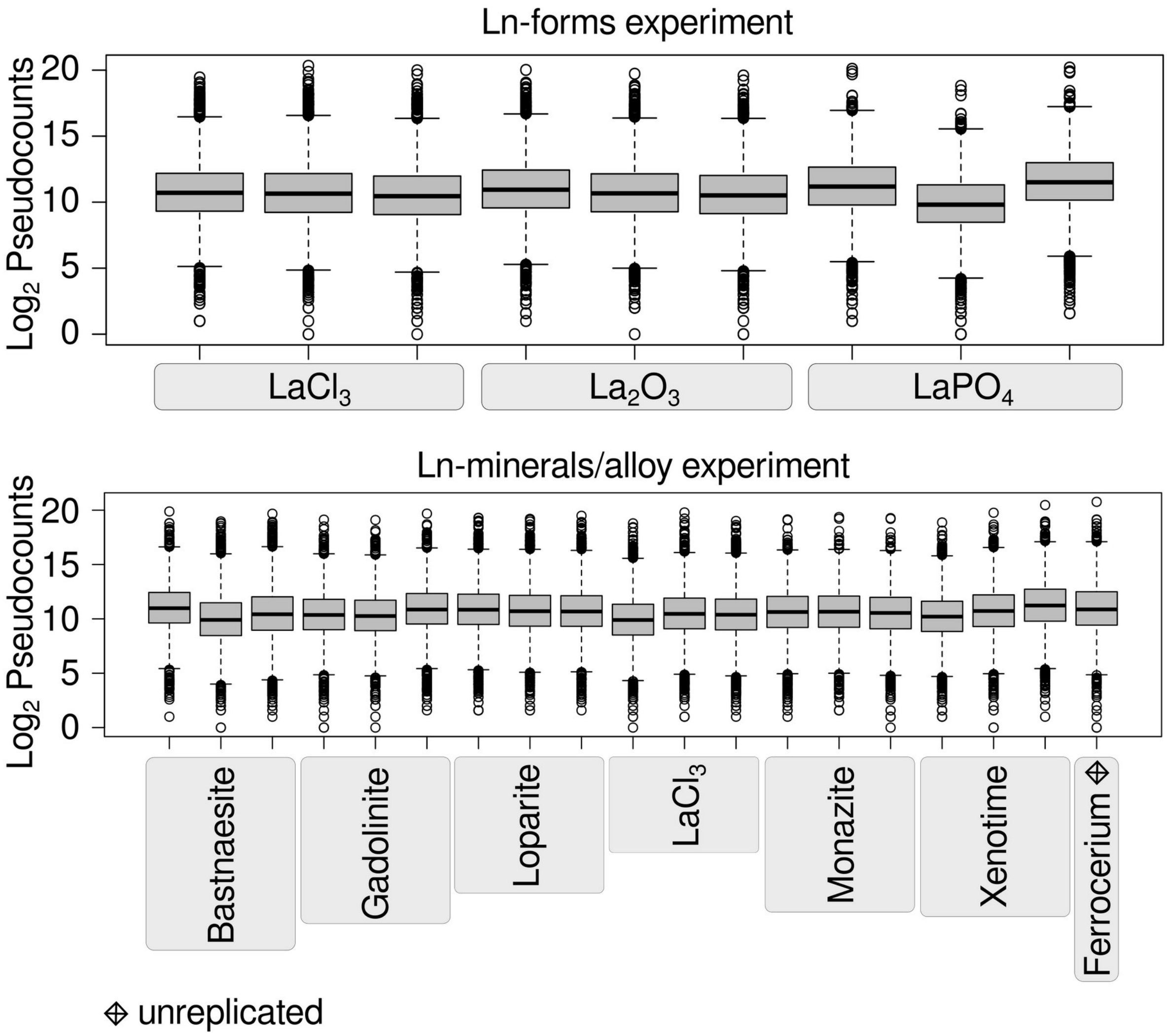
Ln-forms experiment
Log2 Pseudocounts
0
5
10
15
20
LaCl3
La2O3
LaPO4
Ln-minerals/alloy experiment
Log2 Pseudocounts
0
5
10
15
20
Bastnaesite
Gadolinite
Loparite
LaCl3
Monazite
Xenotime
Ferrocerium ⊕
⊕ unreplicated

Figure S2.

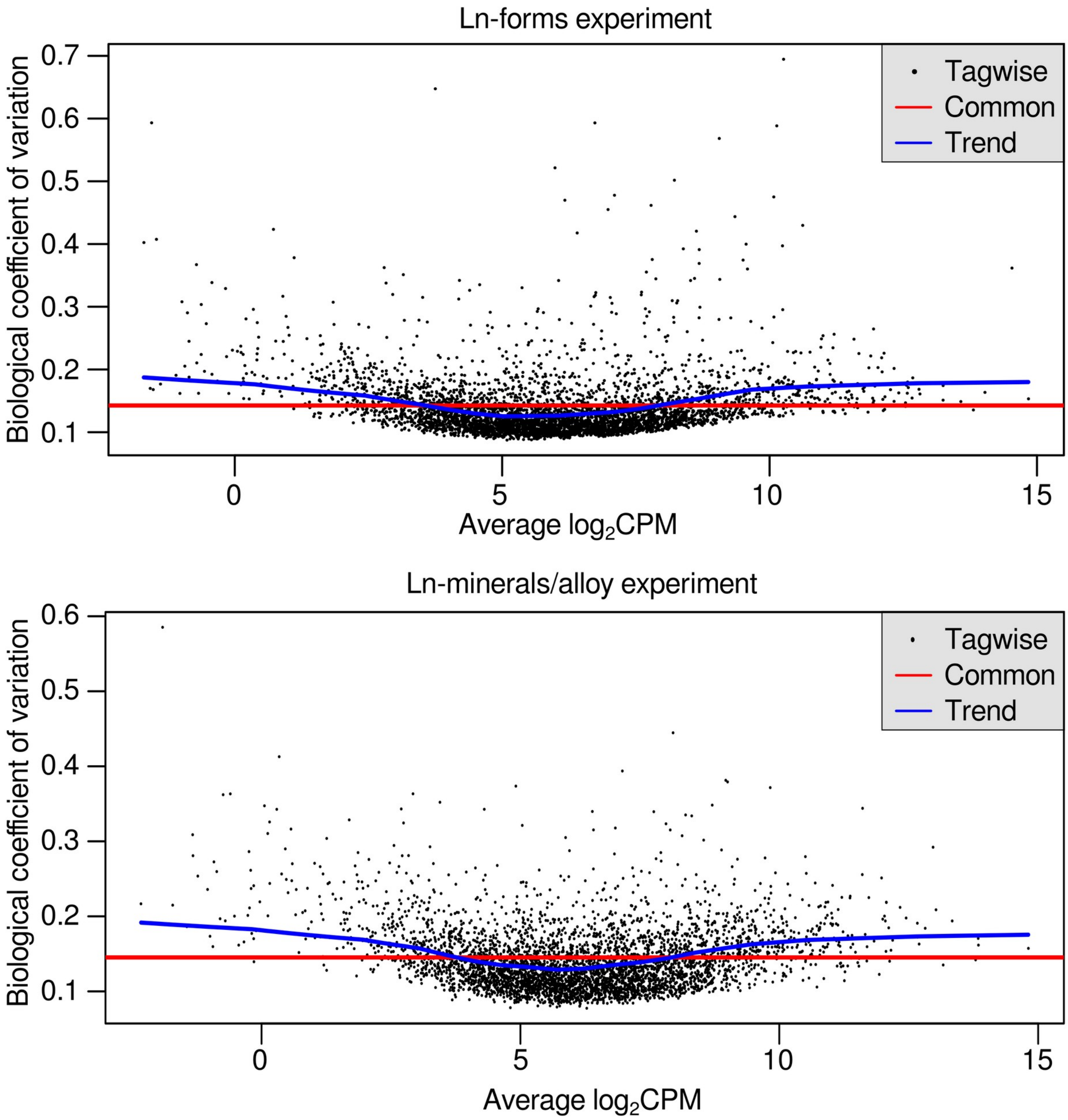
Ln-forms experiment
Biological coefficient of variation
0.1
0.2
0.3
0.4
0.5
0.6
0.7
Average log2CPM
0
5
10
15
Tagwise
Common
Trend
Ln-minerals/alloy experiment
Biological coefficient of variation
0.1
0.2
0.3
0.4
0.5
0.6
Average log2CPM
0
5
10
15
Tagwise
Common
Trend

Figure S3.

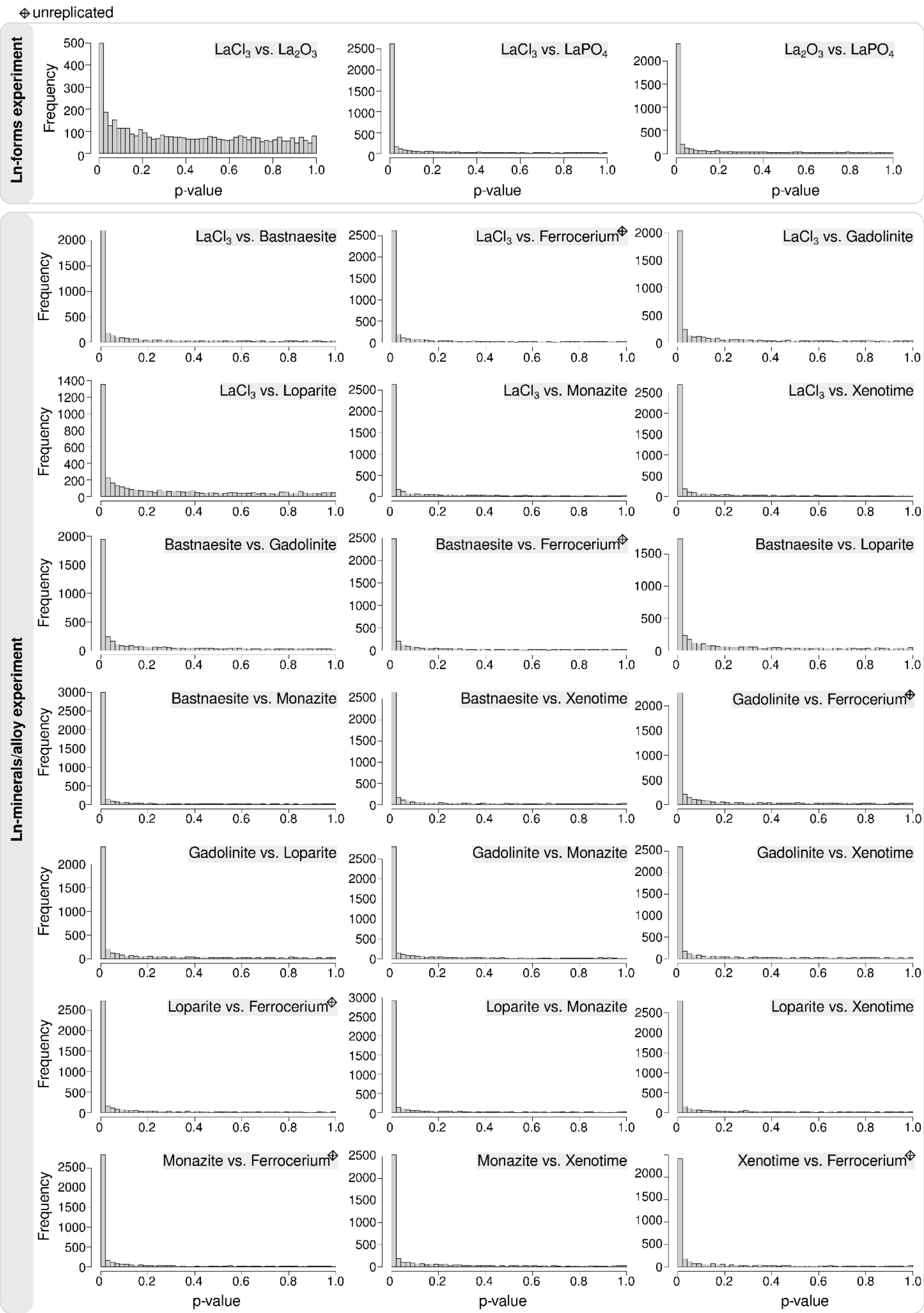
unreplicated
Ln-forms experiment
Ln-minerals/alloy experiment
Frequency
p-value
$LaCl_3$ vs. La_2O_3
$LaCl_3$ vs. $LaPO_4$
La_2O_3 vs. $LaPO_4$
$LaCl_3$ vs. Bastnaesite
$LaCl_3$ vs. Ferrocerium
$LaCl_3$ vs. Gadolinite
$LaCl_3$ vs. Loparite
$LaCl_3$ vs. Monazite
$LaCl_3$ vs. Xenotime
Bastnaesite vs. Gadolinite
Bastnaesite vs. Ferrocerium
Bastnaesite vs. Loparite
Bastnaesite vs. Monazite
Bastnaesite vs. Xenotime
Gadolinite vs. Ferrocerium
Gadolinite vs. Loparite
Gadolinite vs. Monazite
Gadolinite vs. Xenotime
Loparite vs. Ferrocerium
Loparite vs. Monazite
Loparite vs. Xenotime
Monazite vs. Ferrocerium
Monazite vs. Xenotime
Xenotime vs. Ferrocerium

Figure S4.

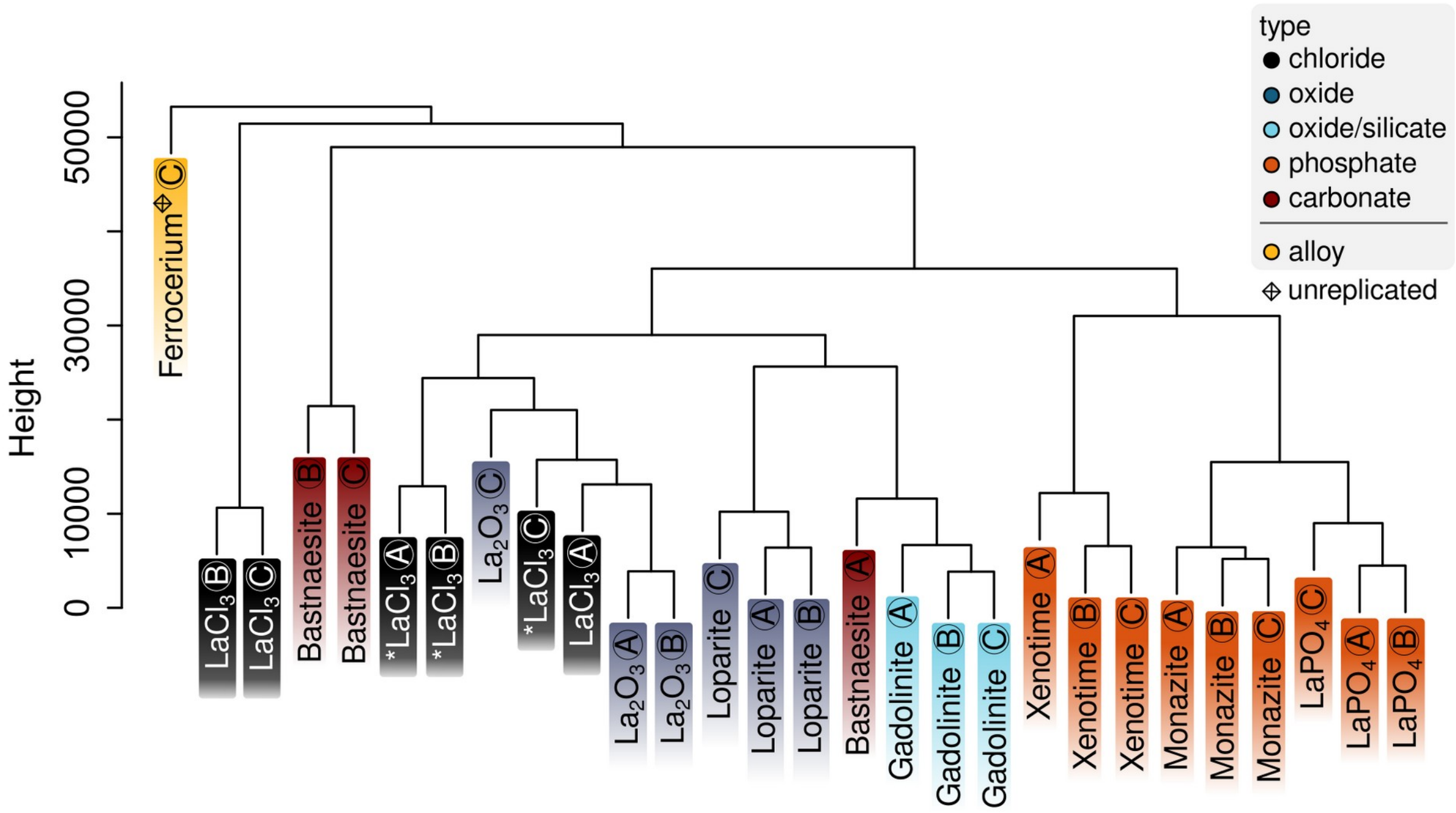
Height
0
10000
30000
50000
type
chloride
oxide
oxide/silicate
phosphate
carbonate
alloy
unreplicated
Ferrocerium C
LaCl3 B
LaCl3 C
Bastnaesite B
Bastnaesite C
*LaCl3 A
*LaCl3 B
La2O3 C
*LaCl3 C
LaCl3 A
La2O3 A
La2O3 B
Loparite C
Loparite A
Loparite B
Bastnaesite A
Gadolinite A
Gadolinite B
Gadolinite C
Xenotime A
Xenotime B
Xenotime C
Monazite A
Monazite B
Monazite C
LaPO4 C
LaPO4 A
LaPO4 B

Figure S5.

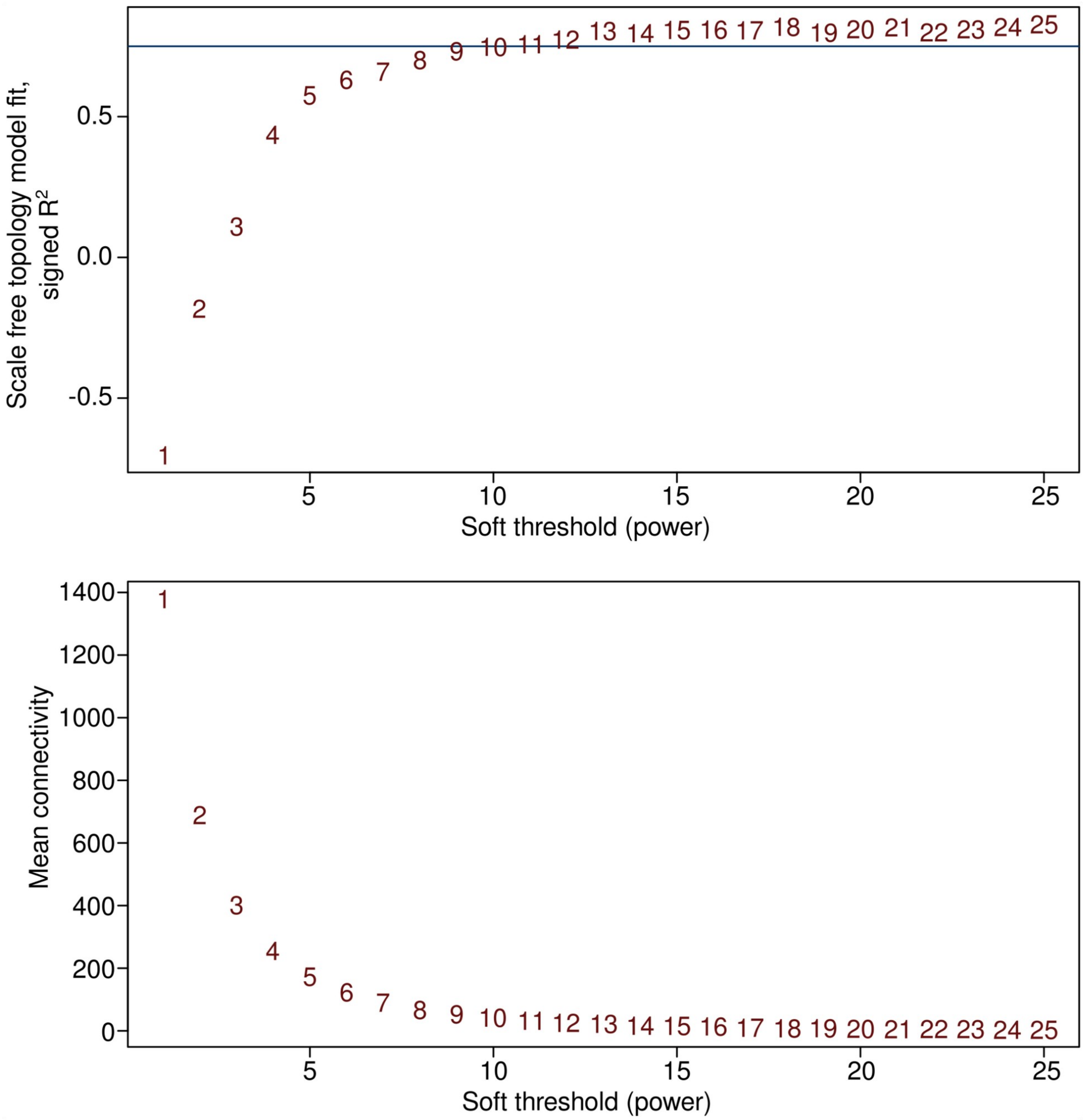
Scale free topology model fit, signed R²
0.5
0.0
-0.5
5
10
15
20
25
Soft threshold (power)
Mean connectivity
1400
1200
1000
800
600
400
200
0
5
10
15
20
25
Soft threshold (power)

Figure S6.

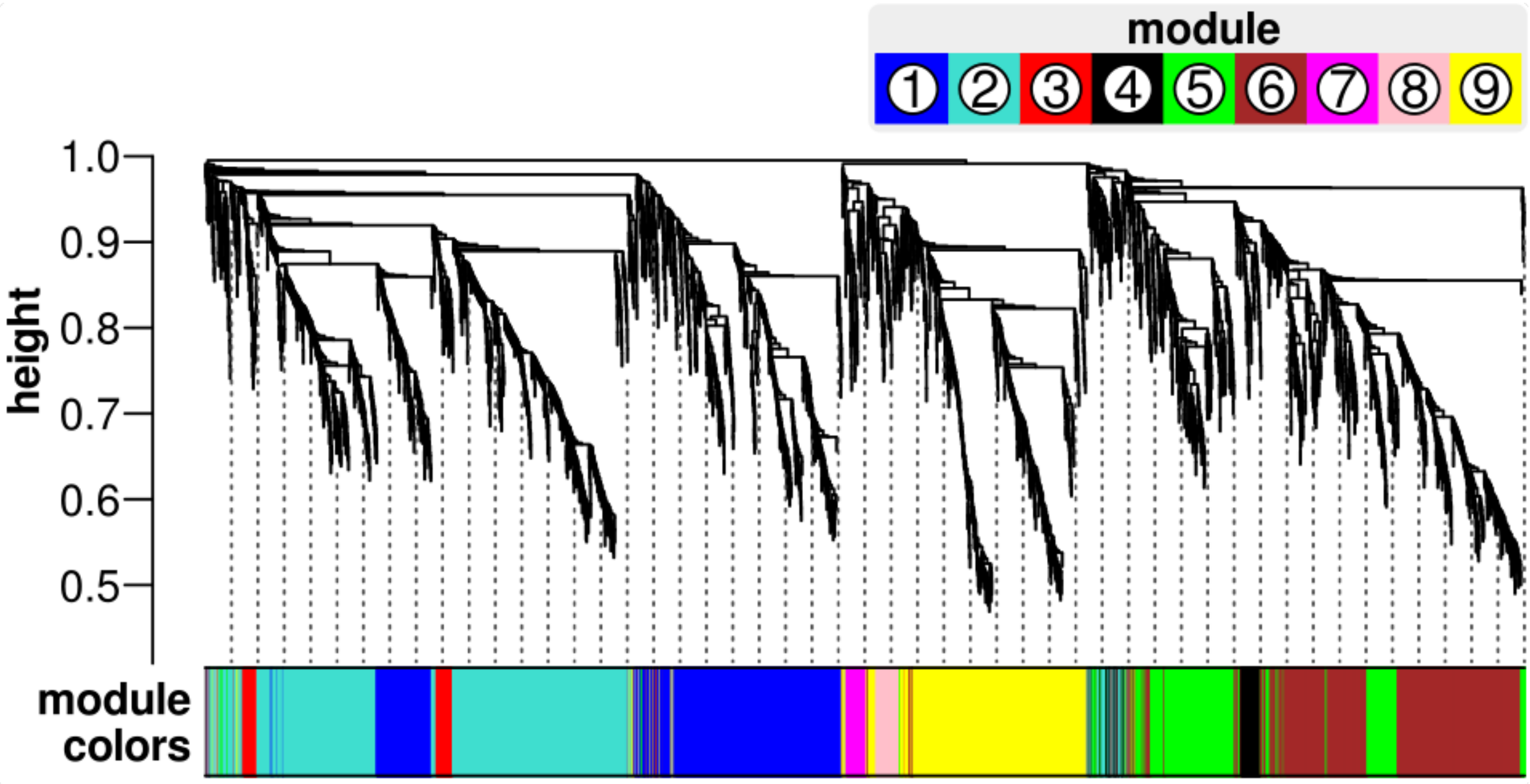

Figure S7.

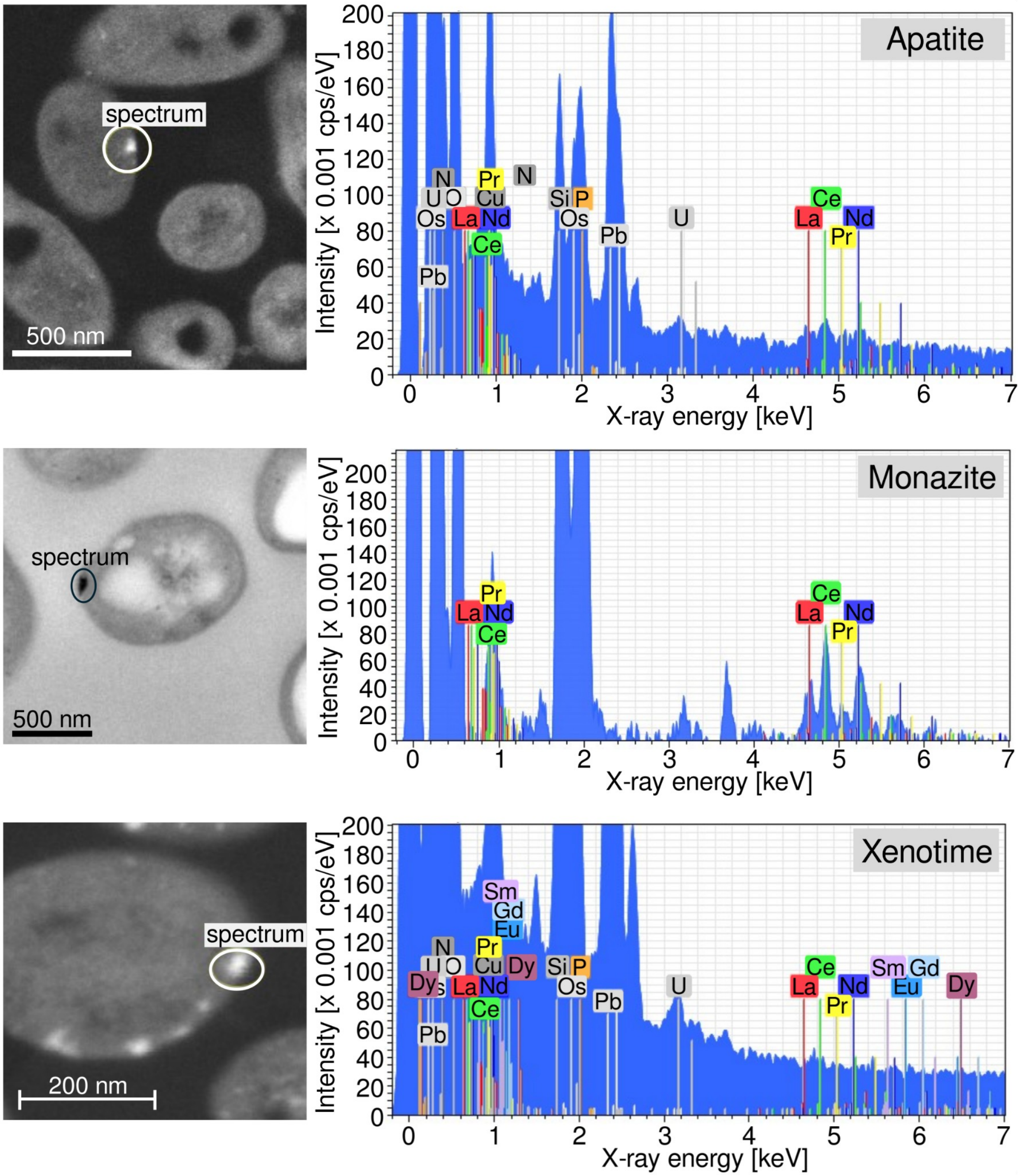
spectrum
500 nm
Apatite
Intensity [x 0.001 cps/eV]
X-ray energy [keV]
N
U O
Os
Pr
Cu
La Nd
Ce
Pb
Si P
Os
Pb
U
Ce
La Nd
Pr
spectrum
500 nm
Monazite
Intensity [x 0.001 cps/eV]
X-ray energy [keV]
Pr
La Nd
Ce
Ce
La Nd
Pr
spectrum
200 nm
Xenotime
Intensity [x 0.001 cps/eV]
X-ray energy [keV]
Sm
Gd
Eu
N
Pr
Cu Dy
La Nd
Ce
Dy
Pb
Si P
Os
Pb
U
Ce
La Nd
Pr
Sm Gd
Eu
Dy

Figure S8.

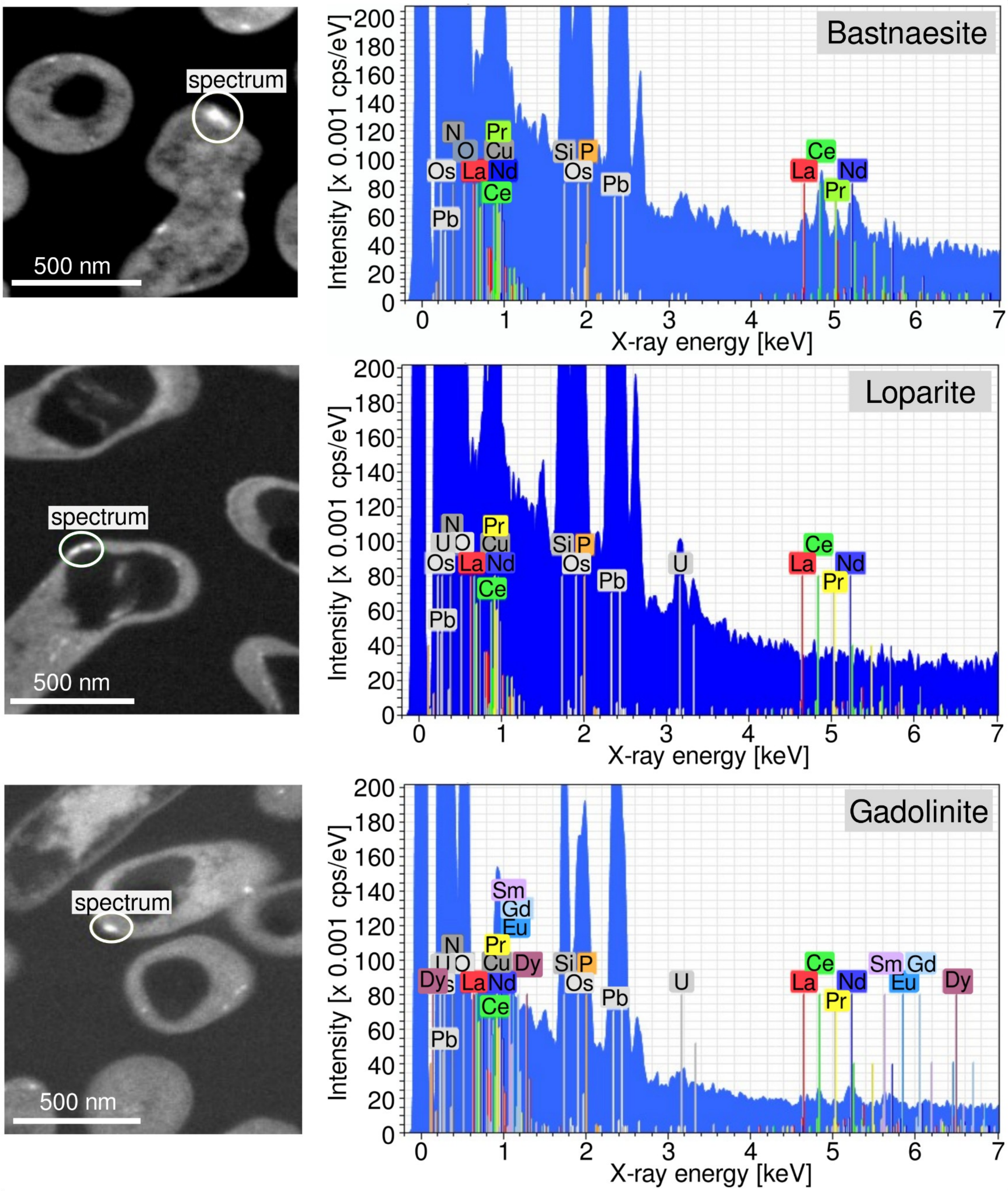
spectrum
500 nm
Bastnaesite
Intensity [x 0.001 cps/eV]
X-ray energy [keV]
N
O
Os
Pb
Pr
Cu
La
Nd
Ce
Si
P
Os
Pb
Ce
La
Nd
Pr
spectrum
500 nm
Loparite
Intensity [x 0.001 cps/eV]
X-ray energy [keV]
N
U
O
Os
Pb
Pr
Cu
La
Nd
Ce
Si
P
Os
Pb
U
Ce
La
Nd
Pr
spectrum
500 nm
Gadolinite
Intensity [x 0.001 cps/eV]
X-ray energy [keV]
Sm
Gd
Eu
N
Pr
U
O
Cu
Dy
Dy
La
Nd
Ce
Pb
Si
P
Os
Pb
U
Ce
La
Nd
Pr
Sm
Gd
Eu
Dy

Figure S9.

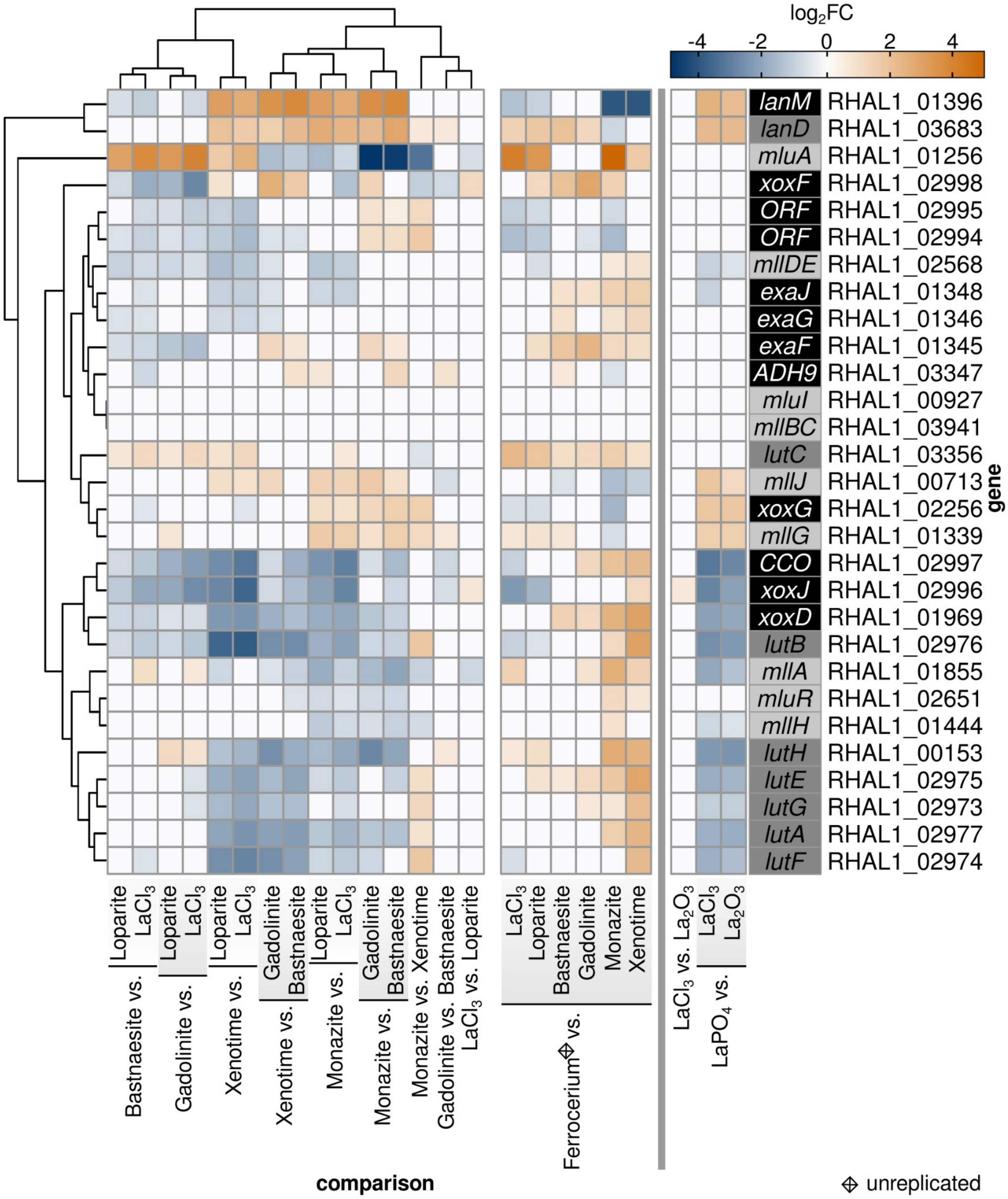
log$_2$FC
-4 -2 0 2 4
lanM RHAL1_01396
lanD RHAL1_03683
mluA RHAL1_01256
xoxF RHAL1_02998
ORF RHAL1_02995
ORF RHAL1_02994
mllDE RHAL1_02568
exaJ RHAL1_01348
exaG RHAL1_01346
exaF RHAL1_01345
ADH9 RHAL1_03347
mluI RHAL1_00927
mllBC RHAL1_03941
lutC RHAL1_03356
mllJ RHAL1_00713
xoxG RHAL1_02256
mllG RHAL1_01339
CCO RHAL1_02997
xoxJ RHAL1_02996
xoxD RHAL1_01969
lutB RHAL1_02976
mllA RHAL1_01855
mluR RHAL1_02651
mllH RHAL1_01444
lutH RHAL1_00153
lutE RHAL1_02975
lutG RHAL1_02973
lutA RHAL1_02977
lutF RHAL1_02974
gene
Bastnaesite vs. Loparite, LaCl$_3$
Gadolinite vs. Loparite, LaCl$_3$
Xenotime vs. Loparite, LaCl$_3$
Xenotime vs. Gadolinite, Bastnaesite
Monazite vs. Loparite, LaCl$_3$
Monazite vs. Gadolinite, Bastnaesite
Monazite vs. Xenotime
Gadolinite vs. Bastnaesite
LaCl$_3$ vs. Loparite
Ferrocerium⊕ vs. LaCl$_3$, Loparite, Bastnaesite, Gadolinite, Monazite, Xenotime
LaCl$_3$ vs. La$_2$O$_3$
LaPO$_4$ vs. LaCl$_3$, La$_2$O$_3$
comparison
⊕ unreplicated

Figure S10.

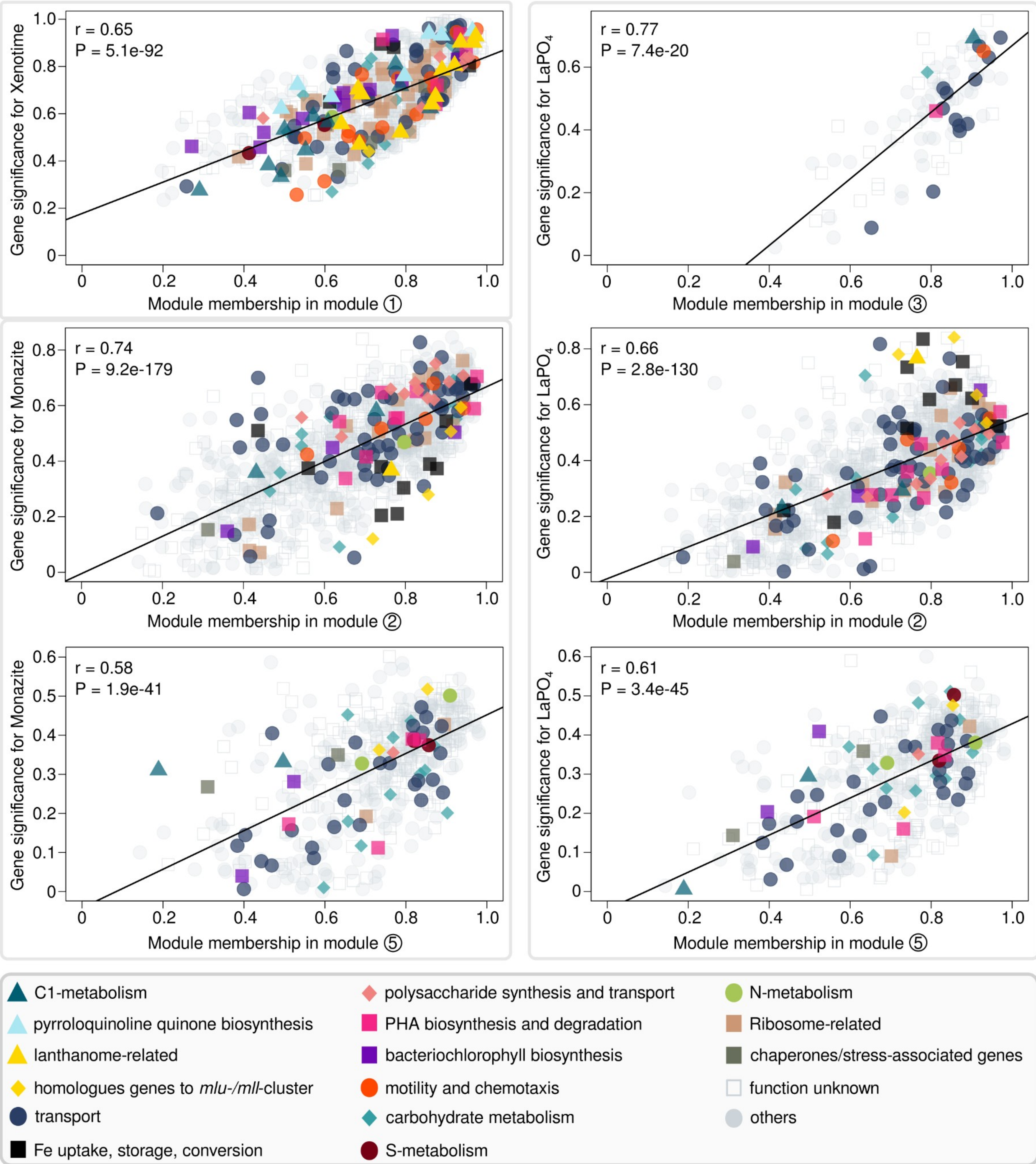
r = 0.65
P = 5.1e-92
Gene significance for Xenotime
Module membership in module ①
r = 0.77
P = 7.4e-20
Gene significance for LaPO$_4$
Module membership in module ③
r = 0.74
P = 9.2e-179
Gene significance for Monazite
Module membership in module ②
r = 0.66
P = 2.8e-130
Gene significance for LaPO$_4$
Module membership in module ②
r = 0.58
P = 1.9e-41
Gene significance for Monazite
Module membership in module ⑤
r = 0.61
P = 3.4e-45
Gene significance for LaPO$_4$
Module membership in module ⑤
C1-metabolism
pyrroloquinoline quinone biosynthesis
lanthanome-related
homologues genes to *mlu-/mll*-cluster
transport
Fe uptake, storage, conversion
polysaccharide synthesis and transport
PHA biosynthesis and degradation
bacteriochlorophyll biosynthesis
motility and chemotaxis
carbohydrate metabolism
S-metabolism
N-metabolism
Ribosome-related
chaperones/stress-associated genes
function unknown
others

Figure S11.

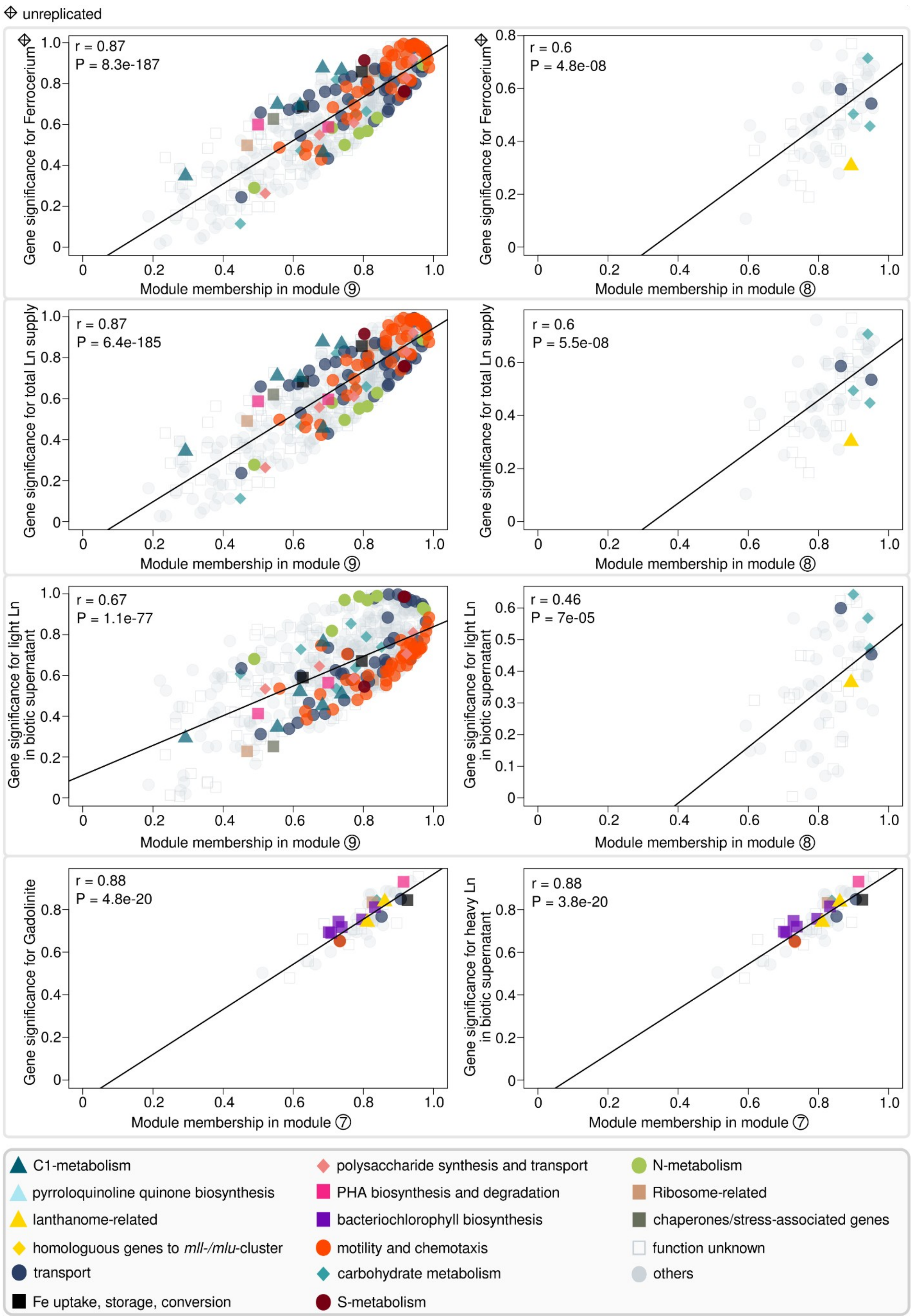

unreplicated
r = 0.87
P = 8.3e-187
Gene significance for Ferrocerium
Module membership in module ⑨
r = 0.6
P = 4.8e-08
Gene significance for Ferrocerium
Module membership in module ⑧
r = 0.87
P = 6.4e-185
Gene significance for total Ln supply
Module membership in module ⑨
r = 0.6
P = 5.5e-08
Gene significance for total Ln supply
Module membership in module ⑧
r = 0.67
P = 1.1e-77
Gene significance for light Ln in biotic supernatant
Module membership in module ⑨
r = 0.46
P = 7e-05
Gene significance for light Ln in biotic supernatant
Module membership in module ⑧
r = 0.88
P = 4.8e-20
Gene significance for Gadolinite
Module membership in module ⑦
r = 0.88
P = 3.8e-20
Gene significance for heavy Ln in biotic supernatant
Module membership in module ⑦
C1-metabolism
pyrroloquinoline quinone biosynthesis
lanthanome-related
homologuous genes to mll-/mlu-cluster
transport
Fe uptake, storage, conversion
polysaccharide synthesis and transport
PHA biosynthesis and degradation
bacteriochlorophyll biosynthesis
motility and chemotaxis
carbohydrate metabolism
S-metabolism
N-metabolism
Ribosome-related
chaperones/stress-associated genes
function unknown
others

Figure S12.

SUPPLEMENTARY TABLES

Table S1. Members of the rare earth elements and the lanthanide series.

Table S2. Acid digestion of ground Ln minerals and ferrocerium in preparation for analytical investigation via ICP-MS was performed using an Anton Paar Microwave 5000. To complex excess HF, a complexation run with boric acid (600 µL boric acid per 100 µL HF) was performed. Following acid digestion, samples were adjusted with water (ultrapure, type 1) to 50 mL volumes. Measurements were performed in sample triplicates and technical triplicates.

Table S3. Overview about the experimental setups including Ln sources, added Ln concentrations and the number of replicates.

Table S4. Ln concentrations and quantities in the used MM2 medium.

Table S5. Enrichment factors (EF) for light, utilizable, heavy Lns regarding lanthanide mobilization (source → supernatant, abiotic and biotic) and uptake/storage (supernatant → deposit). The procedure is outlined in the supplementary material. EFs highlighted in orange were calculated based on lanthanide concentrations in (a)biotic supernatants that were at or below the limit of quantitation (LOQ) of the respective ICP-MS measurements. We substituted these values with LOQ/2. The resulting EFs indicate the direction of enrichment/depletion. They are not quantitative magnitudes. The underlying Ln proportions (light, utilizable, heavy) of the source, abiotic (pH3) supernatant, biotic supernatant, and periplasmic

deposit for each source, from which the EFs were calculated are given as well. These values are rooted in Tables S4, S11, and S13. nc = not calculated.

Table S6. Element-specific lanthanide enrichment factors (EFs) for lanthanide mobilization (source → supernatant, abiotic and biotic) and uptake/storage (supernatant → deposit). The procedure is outlined in the supplementary material. EFs highlighted in orange were calculated based on lanthanide concentrations in (a)biotic supernatants that were at or below the limit of quantitation (LOQ) of the respective ICP-MS measurements. We substituted these values with LOQ/2. The resulting EFs indicate the direction of enrichment/depletion. They are not quantitative magnitudes. nc = not calculated.

Table S7. Permutational multivariate analysis of variance (PERMANOVA). Sequential PERMANOVA partitioning of whole-transcriptome variance (Euclidean distances) among abiotically dissolved Ln concentration [1], iron content of the Ln sources [2], growth yield(carrying capacity) [3], and the identity of the Ln source [4]. Df = degrees of freedom, SumOfSqs = sum of squares, R^2 = proportion of variance explained, F = pseudo-F ratio (mean square of the term/mean square of the residual), Pr(>F) = permutation-based p-value (9999 permutations).

Table S8. Analytical investigation of Ln mineral compositions via energy-dispersive X-ray spectroscopy (EDX). Five replicates per sample were measured using a Helios Nanolab G3 Dual Beam UC (FEI, part of ThermoFisher Scientific, Darmstadt, Germany) with an X-Max 80 SDD detector (Oxford Instruments GmbH, Wiesbaden, Germany).

Table S9. Analytical investigation of Ln mineral compositions via ICP-MS. Measurements were performed in sample triplicates and technical triplicates using an iCAP RQ ICP-MS device (Thermo Fisher Scientific, Frankfurt, Germany). LOD = limit of detection.

Table S10. Growth metrics for each tested Ln source based on logistic growth model parameters from growthcurver. k = carrying capacity, k_se/k_p = standard error/p-value for k, n0 = initial population size, n0_se/n0_p = standard error/p-value for n0, r = growth rate, r_se/r_p = standard error/p-value for r, sigma = residual standard error of the fit, df = degrees of freedom, t_mid = time to reach half of carrying capacity, t_gen = generation (doubling) time, auc_l/auc_e = area under the empirical/fitted logistic curve.

Table S11. Supernatant analytics, determined via ICP-MS. Measurements of selected elements (Ln elements, Be, Ca, Fe, Na, Nb, P, Si, Sr, Ti, Y) were performed with biological triplicates and technical triplicates. Controls without Ln supplementation (MM2 medium with methanol (0.5% v/v, 123 mM), and controls with methanol (0.5% v/v, 123 mM) but no Ln addition were only measured in technical triplicates. SD = standard deviation.

Table S12: Extent of abiotic lanthanide dissolution at pH 3.

Table S13. Investigation of periplasmic Ln accumulations via EDX. The deconvolution analysis of EDX spectra returned the following results. Ln

accumulations were analysed from strain AL1 grown with the Ln minerals apatite, bastnaesite, gadolinite, loparite, monazite, and xenotime.

Table S14. Overview of gene expression data (log_2 counts per million) of strain AL1 in response to different La forms (chloride, oxide, or phosphate, 1 μM). Data were obtained from biological triplicates.

Table S15. Overview of gene expression data (log_2 counts per million) of strain AL1 in response to different Ln minerals (bastnaesite, gadolinite, loparite, monazite, xenotime), ferrocerium, or $LaCl_3$ (1 μM). Data were obtained from biological triplicates except ferrocerium.

Table S16. Overview of the numbers of differentially expressed genes in both experiments. Differentially expressed genes (DEGs) were defined based on a false discovery rate (FDR) < 0.05, log_2 fold changes (log_2FCs) > 0.58 or < (−0.58), and log_2 counts per million (log_2CPM) > 4. DEGs [%] describes the calculated percentages based on the total number of 4323 encoded in the genome of AL1.

Table S17. Matrix containing log_2 fold changes (log_2FCs) of differentially expressed genes, and the number of comparisons in which differential gene expression (DGE) was observed. Empty slots refer to no differential gene expression in the corresponding comparison(s). Gene annotation data was taken from (21).

Table S18. Overview of the gene expression of lanthanome(-related) genes based on log_2CPM values. Genes were grouped as described in the main text. Homologues

for the *mll-/mlu*-cluster from *M. extorquens* AM1, however, showed low homology on the amino acid sequence level (< 30% sequence identity) (21).

Table S19. Overview of differential gene expression of lanthanome-associated genes (log_2FCs). Genes were grouped as described in the main text. Empty slots refer to no differential gene expression in the corresponding comparison(s).

Table S20. Gene clustering based on weighted gene co-expression network analysis. Gene significance (GS) for selected traits and module membership (MM) are listed with corresponding p-values.

REFERENCES

1. Wegner C-E, Westermann M, Steiniger F, Gorniak L, Budhraja R, Adrian L, Küsel K. 2021. Extracellular and Intracellular Lanthanide Accumulation in the Methylotrophic Beijerinckiaceae Bacterium RH AL1. Appl Environ Microbiol 87:e0314420.
2. Gorniak L, Bechwar J, Westermann M, Steiniger F, Wegner C-E. 2023. Different lanthanide elements induce strong gene expression changes in a lanthanide-accumulating methylotroph. Microbiol Spectr 11:e0086723.
3. Sprouffske K, Wagner A. 2016. Growthcurver: an R package for obtaining interpretable metrics from microbial growth curves. BMC Bioinformatics 17:172.
4. Andrews S. 2010. FastQC: a quality control tool for high throughput sequence data. http://www.bioinformatics.babraham.ac.uk/projects/fastqc.
5. Bushnell B. 2016. BBMap short read aligner. https://www.sourceforge.net/projects/bbmap/.
6. Kopylova E, Noé L, Touzet H, Noe L, Touzet H. 2012. SortMeRNA: fast and accurate filtering of ribosomal RNAs in metatranscriptomic data. Bioinformatics 28:3211–3217.
7. Quast C, Pruesse E, Yilmaz P, Gerken J, Schweer T, Yarza P, Peplies J, Glöckner FO. 2013. The SILVA ribosomal RNA gene database project: improved data processing and web-based tools. Nucleic Acids Res 41:D590–6.
8. Yilmaz P, Parfrey LW, Yarza P, Gerken J, Pruesse E, Quast C, Schweer T, Peplies J, Ludwig W, Glöckner FO. 2014. The SILVA and "All-species Living Tree Project (LTP)" taxonomic frameworks. Nucleic Acids Res 42:D643–8.
9. Burge SW, Daub J, Eberhardt R, Tate J, Barquist L, Nawrocki EP, Eddy SR, Gardner PP, Bateman A. 2013. Rfam 11.0: 10 years of RNA families. Nucleic Acids Res 41:D226–32.
10. Li H, Handsaker B, Wysoker A, Fennell T, Ruan J, Homer N, Marth G, Abecasis G, Durbin R, 1000 Genome Project Data Processing Subgroup. 2009. The Sequence Alignment/Map format and SAMtools. Bioinformatics 25:2078–2079.
11. Liao Y, Smyth GK, Shi W. 2013. The Subread aligner: fast, accurate and scalable read mapping by seed-and-vote. Nucleic Acids Res 41:e108.
12. Liao Y, Smyth GK, Shi W. 2014. featureCounts: an efficient general purpose program for assigning sequence reads to genomic features. Bioinformatics 30:923–930.
13. R Core Team. 2022. R: A Language and Environment for Statistical Computing. R Foundation for Statistical Computing, Vienna, Austria.
14. Robinson MD, McCarthy DJ, Smyth GK. 2010. edgeR: a Bioconductor package for differential expression analysis of digital gene expression data. Bioinformatics 26:139–140.
15. Ritchie ME, Phipson B, Wu D, Hu Y, Law CW, Shi W, Smyth GK. 2015. limma powers differential expression analyses for RNA-sequencing and microarray studies. Nucleic Acids Res 43:e47.
16. Rohart F, Gautier B, Singh A, Lê Cao K-A. 2017. mixOmics: An R package for 'omics feature selection and multiple data integration. PLoS Comput Biol 13:e1005752.
17. Rau A, Gallopin M, Celeux G, Jaffrézic F. 2013. Data-based filtering for replicated high-throughput transcriptome sequencing experiments. Bioinformatics 29:2146–2152.
18. Rutter L, Cook D. 2020. bigPint: A Bioconductor visualization package that makes big data pint-sized. PLoS Comput Biol 16:e1007912.
19. Oksanen J, Blanchet FG, Kindt R, Legendre P, Minchin PR, O'Hara RB, Simpson GL, Solymos P, Stevens MHH, Wagner H. 2017. vegan: Community Ecology Package. http://dx.doi.org/10.4135/9781412971874.n145.
20. Robinson MD, Smyth GK. 2008. Small-sample estimation of negative binomial dispersion, with applications to SAGE data. Biostatistics 9:321–332.
21. Wegner C-E, Gorniak L, Riedel S, Westermann M, Küsel K. 2020. Lanthanide-Dependent Methylotrophs of the Family Beijerinckiaceae: Physiological and Genomic Insights. Appl Environ Microbiol 86:e01830–19.
